# Integrating emotion regulation and emotional intelligence traditions: a meta-analysis

**DOI:** 10.3389/fpsyg.2015.00160

**Published:** 2015-02-24

**Authors:** Ainize Peña-Sarrionandia, Moïra Mikolajczak, James J. Gross

**Affiliations:** ^1^Faculty of Psychology, Department of Personality, Evaluation and Psychological Treatments, University of the Basque Country Donostia-San Sebastian, Spain; ^2^Department of Psychology, Research Unit for Emotion Cognition and Health, Université Catholique de Louvain Louvain-la-Neuve, Belgium; ^3^Department of Psychology, Standford University Standford, USA

**Keywords:** emotional intelligence, emotional competence, emotion regulation, coping, review, meta-analysis

## Abstract

Two relatively independent research traditions have developed that address emotion management. The first is the *emotion regulation* (ER) tradition, which focuses on the processes which permit individuals to influence which emotions they have, when they have them, and how they experience and express these emotions. The second is the *emotional intelligence* (EI) tradition, which focuses—among other things—on individual differences in ER. To integrate these two traditions, we employed the process model of ER (Gross, 1998b) to review the literature on EI. Two key findings emerged. First, high EI individuals shape their emotions from the earliest possible point in the emotion trajectory and have many strategies at their disposal. Second, high EI individuals regulate their emotions successfully when necessary but they do so flexibly, thereby leaving room for emotions to emerge. We argue that ER and EI traditions stand to benefit substantially from greater integration.

## Introduction

Contemporary accounts of emotions emphasize the important role they play in adaptation (e.g., Cosmides and Tooby, 2000). Numerous studies support this view, showing that emotions facilitate adaptation by optimizing sensory intake (Susskind et al., 2008; Vermeulen et al., 2009), improving detection of threatening stimuli (e.g., Ohman et al., 2001; Williams et al., 2004; Pessoa et al., 2005), readying behavioral responses (e.g., Frijda, 1987; Roseman et al., 1994), assisting decision making (Damasio, 1994), enhancing memory for important events (Phelps, 2006; Luminet and Curci, 2009), and guiding interpersonal interactions (Keltner and Kring, 1998).

It is also evident, however, that emotions are by no means *always* helpful (e.g., Salovey and Mayer, 1990; Parrott, 1993; Gruber et al., 2011). Indeed, emotions can at times lead us to do very unhelpful things. This is the case, for instance, when anger toward a stubborn administrator only worsens our situation, or when excitement leads us to buy a house that we cannot afford. Emotions may thus be said to be *maladaptive* when they are of the wrong type, when they come at the wrong time, or when they occur at the wrong intensity level. At such times, we often try to *regulate* our emotions (Gross, 1998b).

Because our emotions are crucial determinants of how well we function in everyday life, researchers from different perspectives have energetically sought to understand how emotions best can be managed for optimal functioning. In this article, we focus on two relatively independent research traditions that have examined this question. The first is the emotion regulation (ER) tradition, which has mainly focused on *how* a person can effectively manage his/her emotions. The second is the emotional intelligence (EI) tradition, which has focused—among other things—on understanding *who* makes the most of his/her emotions. Thus far, the ER literature principally has been concerned with basic processes, and only recently has begun to place more emphasis on individual differences. Conversely, the EI literature principally has been concerned with individual differences, and only recently has begun to focus on basic processes. The present paper constitutes an attempt to bring these two traditions together in the hopes that they will both inform and benefit from each other. This effort draws upon prior contributions by Barrett and Gross (2001), Barrett and Salovey (2002), and Matthews et al. (2002), who have all underlined the necessity for the science of emotional intelligence to integrate knowledge acquired in other areas of the affective sciences. This effort is predicated on the belief that EI research can reciprocally inform and complement fundamental research traditions such as ER, first by calling attention to the differences among individuals and second, by emphasizing the consequences of such differences in real life settings (e.g., at work, in education, in marital relationships).

## The emotion regulation (ER) tradition

Emotion regulation refers to the processes by which individuals modify the trajectory of one or more component(s) of an emotional response. Emotion regulation can thus serve to influence the type (i.e., which emotion one has), intensity (i.e., how intense the emotion is), time course (i.e., when the emotion starts and how long it lasts), and quality (i.e., how the emotion is experienced or expressed) of the emotion. Such regulation may be automatic or effortful, conscious or unconscious (Mauss et al., 2006). It occurs every time one (consciously or unconsciously) activates the goal to influence the emotion-generative process (Gross et al., 2011).

Emotion regulation may be intrinsic/intrapersonal (regulating one's own emotions) or extrinsic/interpersonal (regulating someone else's emotions) (Gross and Jazaieri, 2014). In this paper, we will focus on intrapersonal emotion regulation. Although people typically try to decrease the experiential and/or behavioral aspects of negative emotions (Gross et al., 2006), positive emotions are also down-regulated. This is the case when we try to look less happy than we are when we have passed a difficult exam that a friend has failed, when we try to decrease feelings of attraction for a colleague who is married, or when we try to avoid laughing at an inappropriate moment (Giuliani et al., 2008). It is important to note that emotion regulation needn't involve down-regulation. It can also involve maintaining or increasing emotion, as when we maintain enthusiasm in order to achieve a long and difficult task, increase the expression of sadness at a funeral or increase our amusement at a colleague's supposedly funny joke.

The emotion regulation tradition aims to understand the myriad ways individuals regulate their emotions. One critical part of the study of ER has been the conceptual analysis of emotion regulation strategies and the development of a model to organize them. Before presenting this model, it is noteworthy that the ER tradition has learned to avoid classifying ER strategies as *a priori* adaptive or maladaptive. An emotion regulation strategy is said to be contextually adaptive if the resulting emotion meets the regulator's goals, regardless of social norms or long-term adaptive value (Thompson and Calkins, 1996; Gross and Thompson, 2007). If the goal of a boss is to have his subordinates work overtime, his anger up-regulation process will be deemed successful if it results in increased anger and if his subordinates work overtime. The same emotion-regulation strategy can thus be adaptive or maladaptive, depending on the specific individual, the emotion, its intensity, and the context (Bonanno et al., 2004; Sheppes et al., 2011; Aldao and Nolen-Hoeksema, 2012). According to the ER tradition, the adaptive nature of a given emotion-regulation episode is the product of three factors: awareness, goals, and strategies (Gross and Jazaieri, 2014). The awareness of one's emotion and the context in which it occurs makes it possible to determine whether or not the emotion should be regulated (which creates space for flexibility), and to access knowledge about how to do so (Barrett et al., 2001; Farb et al., 2014). The emotion-regulation goal determines whether emotion experience, expression, or physiology must be increased, maintained or decreased in duration and/or intensity. Once the emotion-regulation goal has specified the ends, emotion-regulation strategies specify the means which can be more or less efficient to reach the goal (Gross and Jazaieri, 2014).

**Figure 1:**
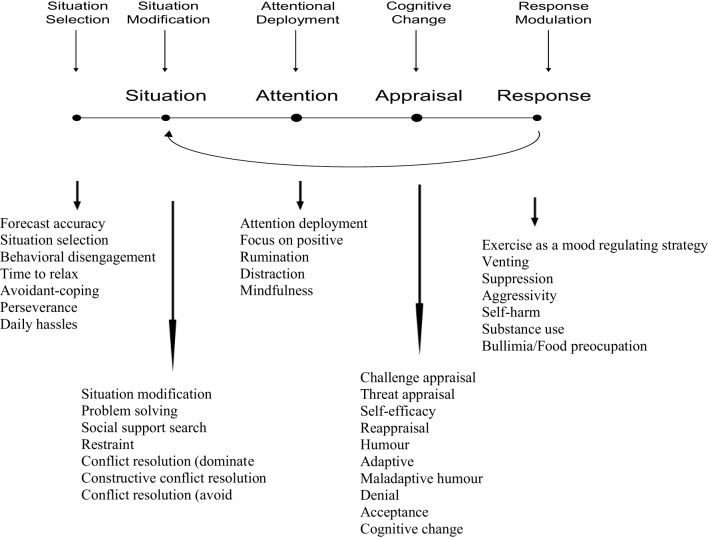
**The process model of emotion regulation (Gross, 1998b, 2014)**.

The process model of emotion regulation (Gross, 1998b) provides a framework for classifying emotion regulation processes regardless of their potential (mal)adaptive value. This model categorizes strategies according to the point at which they have their primary impact in the emotion generative process. During the milliseconds and seconds following the occurrence of a potentially emotion-eliciting situation (micro-level), there are five points in time at which individuals might intervene in order to modify their emotion trajectory. These points represent five families of emotion regulation strategies (see Figure 1). Although sequential at the micro-level, these strategies can be used in parallel or in any order at the macro-level (i.e., at the level of minutes, hours, or days following the emotional situation). Put differently, if the emotion trajectory was not altered or if the emotion was ill-regulated at the micro-level, one can still regulate it later by using any of the five families of strategies.

### Situation selection

Situation selection involves choosing or avoiding some activities, people or places as a function of their expected emotional impact. It is placed at the left-most point in Figure 1 because it affects the situation to which a person is exposed, and thus shapes the emotion trajectory at the earliest possible point. Adaptive situation selection involves knowing oneself and one's needs, forecasting the emotions that various situations are expected to produce (which is not as simple as it may sound; Wilson and Gilbert, 2005), and taking these emotions into account when selecting the situations to which one will be exposed (Loewenstein, 2007; Gross, 2008). Naturally, the costs and benefits in the short and long term must be carefully weighed before making the choice. Two situation selection strategies are confrontation and avoidance.

**Confrontation** involves choosing to face a situation in spite of the negative emotions it might potentially elicit. This strategy is particularly efficient if the situation is likely to bring long-term benefits. Speaking in public often induces negative emotions in the short term, but avoiding oral presentations in front of one's team might turn counterproductive for future promotions. Two meta-analyses indeed confirm that while confrontation often produces negative emotion in the short-term, it is an efficient strategy to maximize long-term happiness and mental health (Suls and Fletcher, 1985; Aldao et al., 2010).

**Avoidance** refers to the escape of the situation as a whole. If a situation is unlikely to bring future benefits and if there are no avoidance-related side effects (or if a situation has more detrimental than beneficial effects), then avoidance is often the best strategy. In the other cases, avoidance is likely to become dysfunctional. Research has shown that the chronic use of avoidance is associated with poor indicators of long-term well-being and health (see Suls and Fletcher, 1985; Penley et al., 2002; Aldao et al., 2010 for meta-analyses and reviews).

Although situation selection may be an efficient strategy, one cannot always avoid negative situations/emotions. The families of ER strategies presented next are useful in three main situations: (1) situations that are expected to induce negative emotions but that cannot be avoided due to positive long-term benefits, (2) unexpected situations that cause an unwanted emotion, and (3) situations that induce a conditioned negative emotion.

### Situation modification

Situation modification, shown in Figure 1, encompasses the strategies aimed at modifying the situation so as to alter its emotional impact. Three strategies have received special attention: direct situation modification, support-seeking, and conflict resolution.

**Direct situation modification** (also called ***problem-focused coping*** in the stress tradition; Folkman and Lazarus, 1980) involves taking practical actions that impact directly on the situation (e.g., fixing the broken printer; rehearsing one's presentation). This strategy is usually associated with increased well-being and less psychological disorders (see Aldao et al., 2010 for a meta-analysis) as well as better health outcomes (see Penley et al., 2002 for a meta-analysis).

**Help/support-seeking** involves seeking others' assistance in modifying the situation (e.g., asking a classmate for some help in order to finish homework by the deadline or seeking the help of a counselor to deal with a difficult child). Although there are situations in which instrumental benefits come at a certain psychological cost (Nadler, 1991), the ability to seek—and obtain—help from others has long been judged adaptive by clinical and educational psychologists (Wills, 1987; Newman, 1994).

**Conflict resolution** involves taking steps to modify (defuse) a conflict situation (e.g., the conflict that my husband and I are having concerning whether to send our daughter to boarding school). While many different strategies can be used (Wall and Callister, 1995), they are not all efficient in reducing conflict (Deutsch et al., 2011). Moreover, while some techniques make it possible to achieve one's ends (e.g., I won: my daughter will stay at home), they can be unsuccessful at addressing the relationship dimensions of the conflict (my husband now agrees with my decision but remains bitter). The opposite could also be true (I may finally agree with my husband but be resentful). Only a few techniques engender satisfaction with both the decision and the relationship (Demoulin, 2014).

Although situation modification strategies make an early impact on the emotion generation process, it is not possible to modify every emotion-eliciting situation: one can neither prevent a sick colleague from coughing loudly every 5 min, nor easily get rid of a tyrannical boss. Other strategies must thus be considered.

### Attentional deployment

Attentional deployment involves altering how we feel by selecting the information we attend to. It comes after situation modification in Figure 1. Existing literature has mainly focused on three forms of attentional deployment: distraction, rumination, and mindfulness.

**Distraction** involves a shift in attention, either away from the situation altogether or away from emotional aspects of the situation. It therefore includes the physical withdrawal (such as covering the eyes in front of a severely injured body) or the internal redirection of attention (such as focusing on the non-emotional aspects of the situation, or thinking about something else). Distraction has been found to decrease negative emotions (see Webb et al., 2012 for a meta-analysis), especially when associated with problem-focused coping (Shimazu and Schaufeli, 2007).

**Rumination** refers to a perseverative focus on thoughts and feelings associated with a negative emotion-eliciting event. It has been found to increase the duration and intensity of negative emotions (Morrow and Nolen-Hoeksema, 1990; Bushman, 2002) and to predict the onset, number and duration of depressive episodes over a 2.5 year follow-up of initially non-depressed individuals (Robinson and Alloy, 2003). Accordingly, this emotion regulation strategy is highly prevalent in clinical populations (see Aldao et al., 2010 for a meta-analysis).

**Mindfulness** (also called “*mindful attention awareness*” to distinguish it from the ritualized practice of mindfulness meditation) involves purposefully paying attention to the present moment in a non-judgmental way. It consists in observing what is happening moment by moment in one's internal (thoughts, motives, emotions, bodily sensations) and external world, without judging it. Numerous cross-sectional, experience-sampling, experimental and intervention studies show that mindful attention increases happiness (Killingsworth and Gilbert, 2010) and decreases negative affects such as stress, anxiety, or depression (Brown and Ryan, 2003; Hofmann et al., 2010). It is worth noting that the balance between internal attention (what I think/feel) and external attention (what I do) is essential. Simply focusing on one's emotions and sensations does not produce the same benefits and can even be counter-productive (see Webb et al., 2012 for a meta-analysis).

### Cognitive change

Cognitive change (shown fourth in line in Figure 1) refers to changing the way we think in order to change the way we feel. We can either change how we think about the situation itself or about our capacity to manage its demands. Like the other strategies, cognitive change can be automatic or effortful. As it is difficult to determine whether the conscious appraisal of a situation is the spontaneous initial appraisal of the situation or the product of an automatic reappraisal, we chose to include both appraisals and re-appraisals in this section. The four forms of (re)appraisals that have received the most attention are self-efficacy appraisals, challenge/threat appraisals, positive reappraisal, and acceptance.

**Self-efficacy** **appraisal** captures an individual's confidence that s/he is able to deal with the situation (Bandura, 1997). Higher levels of self-efficacy lead to both lower subjective stress and increased cellular immunity (e.g., Wiedenfield et al., 1990).

**Challenge and threat appraisals** (Lazarus and Folkman, 1984; Tomaka et al., 1997) refer to the gains and losses perceived in an adverse situation. Threat appraisal is thought to occur when an individual appraises a given situation as exceeding his or her resources and/or focuses on the potential/actual losses inherent to the situation (e.g., loss of love and security in the case of a divorce). By contrast, challenge appraisal occurs in situations appraised as taxing resources but in which the individual, while recognizing the potential or actual losses—which makes it different from unrealistic optimism—focuses on the potential or actual gains inherent to the situation (e.g., increase in autonomy and decrease in quarrels in the case of a divorce). Challenge appraisals lead to less subjective stress and less hypothalamic-pituitary-adrenal axis activation than threat appraisals (e.g., Tomaka et al., 1993; Gaab et al., 2005).

**Positive reappraisal** refers to reappraising a situation or one's response to it in a more positive way. It may consist for instance in looking for the silver lining in the situation, in putting things into perspective or in reinterpreting one's negative emotional response as normal given the circumstances. Studies have shown that reappraisal strategies generally lead to a decrease in negative emotion experience and expression (see Webb et al., 2012 for a meta-analysis). Findings regarding its efficiency to modify physiology are mixed (Webb et al., 2012). While some studies show that reappraisal decreases neuroendocrine and autonomic responses (e.g., Dandoy and Goldstein, 1990; Abelson et al., 2005; Jamieson et al., 2012; Ben-Naim et al., 2013), other studies show that reappraisal increases them (e.g., Denson et al., 2014. Alternative studies have found that reappraisal neither increases nor decreases these responses (e.g., Gross, 1998a). Future studies should uncover the moderators of its effect.

**Acceptance** refers to accepting the situation and/or one's incapacity to deal with it. It is especially useful in situations that cannot be easily modified or reappraised (e.g., abuse as a child; incurable disease). The acceptance of uncontrollable negative events and the emotions that they elicit has been found to be protective at both psychological (decreases negative emotions) and physical (provides immunity and decreases pain) levels (e.g., Burns et al., 2002; McCracken and Eccleston, 2003). This strategy is not frequently used by people suffering from psychological disorders (see Aldao et al., 2010 for a meta-analysis).

### Response modulation

Response modulation is shown on the right side of Figure 1. As this placement indicates, it occurs late in the emotion-generative process, after response tendencies have developed. These strategies can target the experiential (i.e., when one shares one's emotion or drinks a glass of alcohol in order to decrease feelings of anxiety), physiological (i.e., when one smokes marijuana in order to decrease the heart rate, or acts aggressively to defuse physical tension), and/or behavioral (when one attempts to hides one's emotion from others) components of the emotional response. Among response modulation forms, emotion sharing, aggression, substance abuse, and expressive suppression have received the most attention.

**Emotion sharing** refers to expressing one's emotions in a socially shared language (Rimé, 2007). It typically consists in describing the emotional event that one has just experienced or witnessed. If I just saw a pedestrian hit by a car, I will probably call someone I know to tell him or her about it. People share emotion primarily because they expect it to foster emotional recovery (catharsis effect). Research has however shown that sharing *per se* does not foster emotional recovery. Nevertheless, sharing emotion is beneficial to mental health due to several indirect effects such as the construction or reinforcement of social bonds and the transference of affection and warmth (see Rimé, 2007 for a review).

**Verbal/physical aggression** is a strategy used to reduce the bodily tension that arises from an emotional situation (which may or may not be related to the person targeted). Although expressing one's emotions is generally beneficial for both mental and physical health (e.g., Taylor et al., 1997), a number of studies suggest that hostility—and especially its “expressive” dimension—leads to exaggerated cardiovascular reactivity in response to provocative stressors (Suls and Wan, 1993). It also increases the possibility of developing coronary-heart disease (see Miller et al., 1996 for a meta-analysis). Needless to say, this strategy is also very detrimental to social relationships.

**Substance use** refers to exaggerated consumption of alcohol, drugs, or medicines in order to anesthetize thoughts, feelings, and/or physiological arousal. Although moderate alcohol consumption can have health benefits (for a review, see Baum-Baicker, 1985), the regular use (and thus abuse) of alcohol and drugs as a coping style is associated with poor outcomes in terms of mental and physical health (e.g., Single et al., 2000; Teesson et al., 2000).

**Expressive suppression** consists of inhibiting the behavioral expression of unwanted emotions (e.g., hiding one's anger). Research has found that suppression is highly prevalent in several psychological disorders (see Aldao et al., 2010 for a meta-analysis). While it does decrease the observable emotion, suppression rarely changes the negative emotion experience (although it may decrease positive emotion). Moreover, it actually *increases* sympathetic activation of the cardiovascular system (e.g., Gross and Levenson, 1993, 1997; Gross, 1998a; Harris, 2001; Demaree et al., 2006; see Webb et al., 2012 for a meta-analysis). This may explain why expressive suppression decreases well-being (Gross and John, 2003) and increases vulnerability to cardiovascular diseases (see Mauss and Gross, 2004 for review).

Finally, it is worth noting that although it has received little attention, physical exercise can also be used as an emotion regulation strategy.

## The emotional intelligence (EI) tradition

A second tradition that has examined emotion management is the EI tradition. This tradition places emphasis on individual differences rather than on basic processes. It argues that the various instances of emotion regulation are not totally independent of one another within a given individual. On the contrary, individuals show some consistency in their regulation habits (i.e., when, how and which emotion component they regulate). Each individual can thus be characterized by a certain emotion regulation style, which contributes to make him/her predictable in the eyes of others and also carries certain consequences for long-term adaptation (e.g., Bar-On, 1997; Mayer and Salovey, 1997; Gross and John, 2003).

The emotional intelligence (EI) tradition aims to provide a scientific framework for studying individual differences with regard to how individuals identify, understand, express, regulate, and use their own emotions and those of others (Mayer and Salovey, 1997; Petrides and Furnham, 2003; Brasseur et al., 2013). One critical part of the study of EI has been the analysis of individual differences in emotion regulation (Akerjordet and Severinsson, 2007; Roberts et al., 2007). Regardless of the EI model or measure, people scoring high on EI tests are assumed to regulate their emotions better than people scoring low on EI tests.

### Intelligent emotion regulation

The emotional intelligence tradition is outcome-oriented rather than process-oriented in the sense that it seeks to capture the *outcome* of emotion regulation. Individuals are said to display intelligent emotion regulation if they are able to use emotion regulation in a flexible manner and in a way that is consistent with their goals and thus adaptive (Mayer and Salovey, 1995; Bar-On et al., 2003; Mayer et al., 2008). Put differently, emotionally intelligent individuals are those who carefully review the context before deciding whether and how they should regulate their emotion. These individuals take into account and maximize intra-individual and inter-individual long-term survival and welfare. For instance, a boss who up-regulates his anger in order to have his subordinates work overtime will be said to be manipulative but not emotionally intelligent because his emotional behavior will most likely impair his social adaptation in the long-term and lead his subordinates to burn-out. Another feature of emotionally intelligent regulation is that it takes into account the emotional display rules of the culture in which one lives.

In accordance with this adaptation-oriented view, EI has been associated with indicators of superior adaptation in many domains of life. Examples of this are higher life satisfaction (e.g., Petrides et al., 2007b; Di Fabio and Saklofske, 2014b), better health, both objectively measured (Mikolajczak et al., in press) and subjectively reported (see Schutte et al., 2007; Martins et al., 2010 for a meta-analyses), increased social support (e.g., Mikolajczak et al., 2007a), better quality of social and marital relationships (e.g., Schutte et al., 2001a; Lopes et al., 2004; Petrides et al., 2006b; Malouff et al., 2014), and enhanced academic and work performance (see Van Rooy and Viswesvaran, 2004; O'Boyle et al., 2011 for a meta-analysis).

## EI: trait and ability perspectives

Is EI a form of intelligence as has been suggested by authors such as Salovey and Mayer (1990) or is it a constellation of emotion-related traits as authors like Petrides and Furnham (2003) argue? Is it best measured using intelligence-like tests or using personality-like questionnaires? After nearly two decades of debate on the status of EI as intelligence or trait, a tripartite model of EI has been proposed to reconcile these perspectives (see Mikolajczak et al., 2009b).

This model posits three levels of EI: knowledge, abilities, and traits. The knowledge level refers to what people know about emotions and emotional competencies (e.g., *Do I know how* to express my emotions constructively?). The ability level refers to the capability to apply this knowledge in an emotional situation (e.g., *Am I able to* express my emotions constructively?). The focus here is not on what people know but rather on what they do. For instance, even though people know that they should not shout when angry, many are simply unable to refrain from doing so. The trait level refers to emotion-related dispositions, namely, the propensity to behave in a certain way in emotional situations (*Do I typically* express my emotions in a constructive manner?). The focus here is not on what people know or on what they are able to do, but on what they typically do. For instance, some individuals might be able to express their emotions constructively if explicitly asked to do so, but they would be unable to do this spontaneously. These three levels of EI are loosely connected. Knowledge does not always translate into ability, and ability does not always translate into typical behavior. These three levels of EI are therefore assessed using different instruments. Knowledge and ability are assessed using intelligence-like tests such as the MSCEIT^1^, the STEU^2^, or the GERT^3^ while usual emotional behavior is usually assessed using personality-like questionnaires such as the TEIQUE^4^, the EQ-I^5^ or, more recently, the PEC^6^.

Davis and Humphrey (2012) confirmed the construct differentiation and complementary theoretical conceptualizations between ability and trait EI. The findings from a large sample of adolescents revealed that the two measures of EI were only weakly associated. Moreover, each measure showed the expected pattern of associations with personality dimensions (to which trait EI was more robustly associated than ability EI) and general cognitive ability GCA (to which ability EI was more strongly linked than trait EI).

The enthusiasm for the study of emotional intelligence stems in part from its practical implications. Outcomes as different as professional success, well-being, social adjustment, and marital satisfaction could be associated and viewed in a new light. Emotional intelligence indeed predicts a significant portion of variance in these outcomes (see, e.g., O'Boyle et al., 2011 regarding work performance, Schutte et al., 2007 regarding mental health; Mikolajczak et al., in press, for physical health, and Malouff et al., 2014 for marital satisfaction). Partly characterizing these differences as a function of individual differences in emotion regulation processes contributes to explaining—and not simply predicting—these social, health, educational, and occupational outcomes. It also allows researchers and practitioners to identify new avenues for intervention.

## Integrating emotion regulation and emotional intelligence traditions

The ER and EI traditions each capture an important aspect of emotion management. Although the ER tradition has shed light on basic emotion regulation processes, it has not placed emphasis on *individual differences* in these processes. By contrast, the EI tradition has documented the consequences of individual differences in emotion regulation on social, health, educational, and occupational outcomes. However, until recently, EI did not primarily focus on the *processes* generating these individual differences in emotion regulation efficiency. The reason for this is historical: the success of EI in organizational settings has led most EI researchers to focus on the development of measures to improve the quality of EI predictions, rather than on underlying processes.

Our goal in this article is to provide a conceptual map and a review of findings useful to anyone interested in individual differences in emotion regulation processes. In the next section, we will use the process model of emotion regulation described above to organize our review of EI findings in emotion regulation. In each sub-section, the studies reviewed have been meta-analyzed in order to provide an estimate of the effect size reported in the **Tables 2**, **3** (for trait and ability EI, respectively). Two complementary points must be outlined from the outset. First, due to the paucity of EI research in childhood, we have focused on adult literature. Second, several constructs (e.g., social intelligence, alexithymia) have frequently been described as quasi measures of EI. However, as there is a distinction between EI and other related constructs (Van Rooy and Viswesvaran, 2004), the current review only includes studies that use measures that specifically refer to measuring “emotional intelligence.”

### Method

#### Literature search

Relevant studies published until October 2014 were identified using Scopus, PsycINFO, and Pubmed online databases. A combination of the following key words was used: emotional intelligence, emotion regulation, situation selection, situation modification, attention deployment, cognitive change, response modulation, avoidance, forecasting, coping, conflict resolution, problem solving, support seeking, distraction, concentration, rumination, mindfulness, mind wandering, threat appraisal, challenge appraisal, self-efficacy, reappraisal, acceptance, surface acting, suppression, substance use, self-harm. We also wrote to 12 authors of relevant articles and asked for information about unpublished studies between EI and ER (coping). Sixty seven percent of these authors replied but 75% of them did not have unpublished data. We identified four more studies using this method.

#### Inclusion criteria

Studies were included in the review if they met the following criteria:

1. The study presented a correlation between Emotional intelligence (EI) and (at least) one Emotion Regulation (ER) strategy.
2. The sample was constituted of adults.
3. The article was written in English.
4. The sample was constituted of healthy individuals.

Articles were not included if only one subscale of an EI instrument was used (e.g., Gohm and Clore, 2002; Salovey et al., 2002). Additionally, nine studies were not included because we failed to obtain the necessary information from the authors (Pellitteri, 2002; Bond and Donaldson-feilder, 2004; Morrison, 2008; Deniz et al., 2009; Chow, 2011; Frewen et al., 2012; Gawali, 2012; Gooty et al., 2014; Hen and Goroshit, 2014).

Another study was excluded from the meta-analysis because of its unrealistic effect size (Animasahun, 2008 reported a correlation of 0.955 between EI and conflict resolution behavior).

#### Coding studies

The studies were coded with respect to the sample characteristics, the family of emotion regulation strategy based on Gross's process model (i.e., situation selection, situation modification, attentional deployment, cognitive change, and response modulation), the specific ER strategy used, the type of emotional intelligence measure used (ability or trait), the emotional intelligence scale, and the statistical information required to compute the effect size and confidence interval. One researcher coded the relevant articles (A. P.) and another one (M.M.) checked the coding. The two coders settled any disagreement by consensus. A total of 90 studies were coded to produce 200 effect sizes based on 23,174 participants. Twenty five of the effect sizes refer to the situation selection family, 57 effect sizes to the situation modification family, 21 to the attention deployment family, 51 to the cognitive change family, and 46 to the response modulation family. Results are summarized in Table 1.

**Table 1:** **Linking emotional intelligence to the use of emotion regulation strategies**.

**Authors**	***N***	**ER Family**	**Specific variable**	**T/A**	**Measure used**	**Facet**	***r***	**CI *r* (lower bound)**	**CI *r* (upper bound)**	***d***
Schutte et al., 2009	73	SS	Situation selection	T	AES		0.30	0.08	0.49	0.63
Ciarrochi et al., 2002	302	SS	Daily hassles	T	EIS		−0.01	−0.12	0.10	−0.02
Day et al., 2005	133	SS	Daily hassles	T	EQ-I	AGS	−0.43	−0.51	−0.27	−0.94
Kim and Agrusa, 2011	385	SS	Avoidant coping	T	WLEIS		0.15	0.05	0.25	0.30
Shah and Thingujam, 2008	197	SS	Avoidant coping	T	EIS	AGS	−0.08	−0.22	0.06	−0.16
Rogers et al., 2006	253	SS	Avoidant coping	T	SREIT		−0.04	−0.16	0.08	−0.08
Velasco et al., 2006	593	SS	Avoidant coping	T	TMMS-alex F1		−0.16	−0.24	−0.08	−0.32
Petrides et al., 2007a (study 1)	166	SS	Avoidant coping	T	modified EQ-i		−0.34	−0.47	−0.20	−0.72
Petrides et al., 2007a (study2 sample1)	200	SS	Avoidant coping	T	TEIQue-LF		−0.39	−0.50	−0.27	−0.84
Petrides et al., 2007b	274	SS	Avoidant coping	T	TEIQue-LF		0.01	−0.11	0.13	0.02
MacCann et al., 2011	159	SS	Avoidant coping	A	MSCEIT	AGS	−0.21	−0.35	−0.06	−0.43
Gerits et al., 2004	380	SS	Avoidant coping	T	EQ-i		−0.23	−0.32	−0.13	−0.47
Monaci et al., 2013	198	SS	Avoidant coping	T	SEIS		0.01	−0.13	0.15	0.02
Mikolajczak et al., 2009a	490	SS	Avoidant coping	T	TEIQue-ASF		−0.26	−0.34	−0.18	−0.54
Petrides et al., 2006a	37	SS	Perseverance	T	TEIQue-LF		0.53	0.25	0.74	1.25
Dunn et al., 2007	84	SS	Forecast accuracy	A	MSCEIT		−0.19	0.06	0.62	−0.38
Dunn et al., 2007	84	SS	Forecast accuracy	T	SREIS		−0.09	−0.39	0.02	−0.18
Hoerger et al., 2012 (study 1)	81	SS	Forecast accuracy	T	SEI/SREIS/TEIQue		0.22	−0.30	0.13	0.45
Hoerger et al., 2012 (study 1)	81	SS	Forecast accuracy	A	JET+IJI		0.36	−0.01	0.41	0.77
Hoerger et al., 2012 (study 2)	81	SS	Forecast accuracy	T	TEIQue-SF		0.27	0.16	0.54	0.56
Hoerger et al., 2012 (study 2)	81	SS	Forecast accuracy	A	JET+IJI+STEU		0.39	0.07	0.47	0.84
Schutte et al., 2001b	38	SS	Persistence	T	SEIS		0.37	0.19	0.56	0.79
Bastian et al., 2005	246	SS	Behavioral disengagement	T	TMMS+AES	R+C	−0.24	−0.35	−0.12	−0.49
Bastian et al., 2005	246	SS	Behavioral disengagement	A	MSCEIT		−0.16	−0.28	−0.04	−0.32
Tsaousis and Nikolaou, 2005	365	SS	Time to relax	T	TEIQ		0.43	0.35	0.51	0.95
Schutte et al., 2009	73	SM	Modifying situations	T	EIS		0.20	−0.03	0.42	0.41
Moradi et al., 2011	200	SM	Problem-focused coping	T	TMMS	R+C	0.27	0.14	0.40	0.56
Petrides et al., 2007a (study 1)	166	SM	Problem-focused coping	T	modified EQ-i		0.57	0.46	0.67	1.38
Petrides et al., 2007a (study 2. sample 1)	200	SM	Problem-focused coping	T	TEIQue-LF		0.67	0.59	0.75	1.80
Petrides et al., 2007b	274	SM	Problem-focused coping	T	TEIQue-LF		0.64	0.57	0.71	1.66
MacCann et al., 2011	159	SM	Problem-focused coping	A	MSCEIT	AGS	0.14	−0.02	0.29	0.28
Shah and Thingujam, 2008	197	SM	Problem-focused coping	T	EIS	AGS	0.21	0.07	0.34	0.43
Goldenberg et al., 2006	223	SM	Problem-focused coping	T	SREIS		0.55	0.46	0.64	1.31
Goldenberg et al., 2006	223	SM	Problem-focused coping	A	MSCEIT		0.17	0.04	0.30	0.34
Kluemper, 2008	180	SM	Problem-focused coping	T	WLEIS		0.61	0.51	0.70	1.54
Rogers et al., 2006	253	SM	Problem-focused coping	T	SREIT		0.30	0.19	0.41	0.63
Mikolajczak et al., 2008	203	SM	Problem-focused coping	T	TEIQue-LF		0.40	0.29	0.51	0.87
Salovey et al., 2002 (study 3)	48	SM	Problem-focused coping	T	TMMS	R+C	0.34	0.06	0.57	0.71
Saklofske et al., 2007	362	SM	Problem-focused coping	T	EIS		0.38	0.29	0.46	0.82
Almran and Punamaki, 2008	312	SM	Problem-focused coping	T	EQ-I:YV-S		0.25	0.14	0.36	0.51
Velasco et al., 2006	593	SM	Problem-focused coping	T	TMMS-alex F1		0.20	0.12	0.28	0.41
Rahim and Minors, 2003	222	SM	Problem solving	T	EQ-Index	AGS	0.44	0.33	0.54	0.98
Monaci et al., 2013	198	SM	Direct confrontation	T	SEIS		0.43	0.31	0.54	0.95
Montes-Berges and Augusto, 2007	119	SM	Active coping	T	TMMS-24	R+C	0.05	−0.13	0.23	0.11
Gerits et al., 2004	380	SM	Active coping	T	EQ-I		0.38	0.29	0.46	0.82
Tsarenko and Strizhakova, 2013	252	SM	Active coping	T	SREIS		0.45	0.34	0.54	1.01
Zomer, 2012	300	SM	Active coping	T	TMMS-24	R+C	0.15	0.04	0.26	0.30
Bastian et al., 2005	246	SM	Active coping + planning	T	TMMS+AES	R+C	0.40	0.28	0.51	0.87
Bastian et al., 2005	246	SM	Active coping + planning	A	MSCEIT		0.05	−0.08	0.18	0.10
Bastian et al., 2005	246	SM	Problem Solving Inventory	T	TMMS+AES	R+C	−0.43	−0.53	−0.32	−0.94
Bastian et al., 2005	246	SM	Problem Solving Inventory	A	MSCEIT		−0.04	−0.17	0.09	−0.08
Austin et al., 2010	475	SM	Task-oriented coping	T	EQ:i-S	AGS	0.38	0.30	0.45	0.82
Saklofske et al., 2012	238	SM	Task-oriented coping	T	EQ:i S	AGS	0.48	0.38	0.57	1.09
Kim and Agrusa, 2011	385	SM	Task coping	T	WLEIS		0.54	0.46	0.61	1.28
Mikolajczak et al., 2009a	490	SM	Rational coping	T	TEIQue-ASF		0.46	0.39	0.53	1.03
Monaci et al., 2013	198	SM	Social support	T	SEIS		0.36	0.24	0.48	0.77
Bastian et al., 2005	246	SM	Instrumental social support	T	TMMS+AES	R+C	0.24	0.11	0.35	0.49
Bastian et al., 2005	246	SM	Instrumental social support	A	MSCEIT		0.06	−0.07	0.19	0.12
Zomer, 2012	300	SM	Support from others	T	TMMS-24	R+C	0.11	0.00	0.22	0.21
Gerits et al., 2004	380	SM	Social support seeking	T	EQ-i		0.21	0.11	0.31	0.43
Shah and Thingujam, 2008	197	SM	Social support seeking	T	EIS	AGS	0.07	−0.08	0.21	0.13
Ciarrochi and Deane, 2001	300	SM	Social support seeking	T	EIS	AGS	0.15	0.05	0.25	0.26
Velasco et al., 2006	593	SM	Social support seeking	T	TMMS-alex F1		0.13	0.05	0.21	0.26
Goldenberg et al., 2006	223	SM	Social support seeking	T	SREIS		0.32	0.20	0.43	0.67
Goldenberg et al., 2006	223	SM	Social support seeking	A	MSCEIT		0.04	−0.09	0.17	0.08
Zeidner and Kloda, 2013	200	SM	Conflict res. (constructive)	A	MSCEIT		0.24	0.11	0.37	0.49
Zeidner and Kloda, 2013	200	SM	Conflict res. (avoidance)	A	MSCEIT		−0.39	−0.50	−0.26	−0.84
Jordan and Troth, 2004	350	SM	Conflict res. (integrate)	T	WEIP6		0.35	0.25	0.44	0.74
Jordan and Troth, 2004	350	SM	Conflict res. (avoid)	T	WEIP6		−0.12	−0.23	−0.01	−0.24
Jordan and Troth, 2004	350	SM	Conflict res. (dominate)	T	WEIP6		0.19	0.09	0.29	0.38
Jordan and Troth, 2002	139	SM	Conflict res. (collaborate)	T	WEIP6		0.53	0.40	0.64	1.25
Jordan and Troth, 2002	139	SM	Conflict res. (avoidance)	T	WEIP6		−0.12	−0.28	0.04	−0.24
Jordan and Troth, 2002	139	SM	Conflict resolution (force)	T	WEIP6		0.02	−0.15	0.19	0.04
Jordan and Troth, 2002	139	SM	Conflict res. (accommodate)	T	WEIP6		−0.08	−0.24	0.08	−0.01
Jordan and Troth, 2002	139	SM	Conflict res. (compromise)	T	WEIP6		0.09	−0.08	0.25	0.02
Salami, 2010b	320	SM	Conflict res. (confronting)	T	WLEIS		0.20	0.10	0.30	0.41
Salami, 2010b	320	SM	Conflict res. (withdrawal)	T	WLEIS		0.12	0.01	0.23	0.24
Salami, 2010b	320	SM	Conflict resolution (forcing)	T	WLEIS		0.12	0.01	0.23	0.24
Salami, 2010b	320	SM	Conflict res. (smoothe)	T	WLEIS		0.19	0.08	0.29	0.38
Salami, 2010b	320	SM	Conflict res. (compromise)	T	WLEIS		0.21	0.10	0.31	0.43
Bastian et al., 2005	246	SM	Restraint	T	TMMS+AES		0.08	−0.05	0.21	0.16
Bastian et al., 2005	246	SM	Restraint	A	MSCEIT		−0.04	−0.17	0.09	−0.08
Schutte et al., 2009	73	AD	Attention deployment	T	EIS		0.38	0.17	0.56	0.82
Bastian et al., 2005	246	AD	Mental disengagement	T	TMMS+AES		−0.05	−0.18	0.08	−0.10
Bastian et al., 2005	246	AD	Mental disengagement	A	MSCEIT		−0.05	−0.18	0.08	−0.10
Mikolajczak et al., 2008	203	AD	Trait distraction	T	TEIQue-LF		0.41	0.29	0.52	0.89
Salovey et al., 2002 (study 3)	48	AD	State distraction	T	TMMS	R+C	0.36	0.08	0.59	0.77
Saklofske et al., 2012	238	AD	Distraction	T	EQ-i: S		−0.11	−0.23	0.02	−0.21
Austin et al., 2010	475	AD	Distraction	T	EQ-i: S		0.18	0.09	0.27	0.37
Lanciano et al., 2012	157	AD	Dysfunctional rumination	A	MSCEIT	AGS	−0.44	−0.56	−0.30	−0.98
Petrides et al., 2007b	274	AD	Trait rumination	T	TEIQue-LF		−0.47	−0.56	−0.38	−1.06
Petrides et al., 2007a (study 1)	166	AD	Trait rumination	T	modified EQ-i		−0.53	−0.64	−0.42	−1.25
Mikolajczak et al., 2008	203	AD	Trait Rumination	T	TEIQue-LF		−0.10	−0.24	0.04	−0.20
Ramos et al., 2007	144	AD	State rumination	T	TMMS	R+C	−0.11	−0.27	0.05	−0.21
Salovey et al., 2002 (study 3)	48	AD	State rumination	T	TMMS	R+C	−0.27	−0.51	0.02	−0.55
Salguero et al., 2013	1154	AD	Rumination	T	TMMS-24	R+C	−0.11	−0.17	−0.05	−0.22
Brown and Ryan, 2003	645	AD	Mindful attention	T	TMMS-24	R+C	0.40	0.33	0.46	0.87
Wang and Kong, 2014	321	AD	Mindful attention	T	WLEIS		0.33	0.23	0.42	0.70
Schutte and Malouff, 2011	125	AD	Mindful attention	T	AES		0.65	0.53	0.74	1.71
Kokinda, 2010	108	AD	Mindful attention	T	AES		0.47	0.31	0.63	1.06
Charoensukmongkol, 2014	317	AD	Mindful attention	T	WLEIS		0.32	0.22	0.42	0.68
Baer et al., 2004 (study 4)	130	AD	Mindful attention	T	TMMS-24	R+C	0.24	0.07	0.40	0.49
Totterdell and Holman, 2003	18	AD	Focus on positive	T	EIS		0.46	−0.01	0.79	1.03
Schutte et al., 2009	73	CC	Cognitive change	T	AES		0.26	0.03	0.46	0.54
Charoensukmongkol, 2014	317	CC	Self-efficacy	T	WLEIS		0.68	0.61	0.74	1.86
Brown et al., 2003	288	CC	Self-efficacy	T	EII-R		0.25	0.14	0.36	0.50
Chan, 2004	158	CC	Self-efficacy	T	EIS		0.32	0.17	0.45	0.66
Martin et al., 2004	140	CC	Self-efficacy	T	EJI		0.54	0.41	0.65	1.27
Durán et al., 2006	373	CC	Self-efficacy	T	TMMS	R+C	0.34	0.24	0.42	0.72
Kaur et al., 2006	117	CC	Self-efficacy	T	EIS		0.42	0.26	0.56	0.92
Villanueva and Sanchez, 2007	70	CC	Self-efficacy	T	SSRI		0.36	0.13	0.54	0.77
Di Fabio and Palazzeschi, 2008	169	CC	Self-efficacy	T	EQ-i		0.34	0.20	0.47	0.72
Adeyemo, 2007	300	CC	Self-efficacy	T	EQ-i		0.17	0.06	0.28	0.34
Mikolajczak et al., 2006	95	CC	Self-efficacy	T	TEIQue-LF		0.66	0.53	0.76	1.75
Mikolajczak and Luminet, 2008 (study1)	27	CC	Self-efficacy	T	TEIQue-LF		0.50	0.15	0.75	1.15
Mikolajczak and Luminet, 2008 (study2)	15	CC	Self-efficacy	T	TEIQue-SF		0.29	−0.27	0.70	0.60
Kirk et al., 2008	207	CC	Self-efficaccy	T	AES		0.73	0.66	0.79	2.13
Kirk et al., 2008	207	CC	Self-efficaccy	A	MSCEIT		0.34	0.22	0.46	0.72
Animasahun, 2008	300	CC	Self-efficacy	T	EIS		0.43	0.33	0.52	0.95
Salami, 2010a	242	CC	Self-efficacy	T	WLEIS		0.08	−0.05	0.21	0.16
Tsarenko and Strizhakova, 2013	252	CC	Self-efficacy	T	SREIS		0.57	0.48	0.65	1.38
Mouton et al., 2013	119	CC	Self-efficacy	T	TEIQue		0.28	0.11	0.44	0.58
Di Fabio and Saklofske, 2014b	164	CC	Core self-evaluation^a^	T	TEIQue/EQ-i		0.56	0.45	0.65	1.35
Di Fabio and Saklofske, 2014b	164	CC	Core self-evaluation	A	MSCEIT		0.02	−0.13	0.17	0.04
Kluemper, 2008	180	CC	Core self-evaluation	T	WLEIS		0.73	0.65	0.79	2.14
Di Fabio and Saklofske, 2014a	194	CC	Self-Efficacy	A	MSCEIT		0.23	0.09	0.35	0.47
Di Fabio and Saklofske, 2014a	194	CC	Self-Efficacy	T	EQ-i		0.67	0.58	0.74	1.80
Mikolajczak and Luminet, 2008 (study2)	15	CC	Ratio challenge/threat	T	TEIQue-SF		0.72	0.33	0.9	2.07
Mikolajczak et al., 2006	70	CC	Challenge appraisal	T	TEIQue-LF		0.02	−0.22	0.26	0.04
Mikolajczak et al., 2006	70	CC	Threat appraisal	T	TEIQue-LF		−0.41	−0.60	−0.20	−0.89
Schutte et al., 2009	73	CC	Reappraisal	T	AES		0.46	0.26	0.62	1.03
Shah and Thingujam, 2008	197	CC	Positive reappraisal	T	EIS	AGS	0.21	0.07	0.34	0.43
Mikolajczak et al., 2008	203	CC	Reappraisal	T	TEIQue-LF		0.46	0.34	0.56	1.03
Velasco et al., 2006	593	CC	Positive reappraisal	T	TMMS-alex F1		0.23	0.15	0.31	0.47
Kafetsios and Loumakou, 2007	475	CC	Reappraisal	T	EQ-I		0.04	−0.08	0.16	0.08
Cabello et al., 2013	866	CC	Reappraisal	T	TMMS-24	R+C	0.34	0.28	0.40	0.72
Coumans, 2005	31	CC	Reappraisal	T	TEIQue-LF		0.02	−0.34	0.38	0.04
Totterdell and Holman, 2003	18	CC	Perspective taking	T	EIS		0.21	−0.29	0.62	0.43
Moradi et al., 2011	200	CC	Reappraisal	T	TMMS	R+C	0.45	0.34	0.56	1.01
Bastian et al., 2005	246	CC	Positive interpretation	T	TMMS+AES	R+C	0.50	0.40	0.59	1.15
Bastian et al., 2005	246	CC	Positive interpretation	A	MSCEIT		0.11	−0.02	0.24	0.21
Bastian et al., 2005	246	CC	Adaptive Humour	T	TMMS+AES	R+C	0.14	0.01	0.26	0.28
Bastian et al., 2005	246	CC	Adaptive Humour	A	MSCEIT		0.04	−0.09	0.17	0.08
Zomer, 2012	300	CC	Adaptive Humour	T	TMMS-24	R+C	0.01	−0.10	0.12	0.02
Greven et al., 2008	1038	CC	Adaptive Humour	T	TEIQue-LF		0.45	0.40	0.50	0.99
Greven et al., 2008	1038	CC	Maladaptive Humour	T	TEIQue-LF		−0.27	−0.33	−0.21	−0.55
Tsarenko and Strizhakova, 2013	252	CC	Denial	T	SREIS		−0.09	−0.21	0.03	−0.18
Zomer, 2012	300	CC	Denial	T	TMMS-24	R+C	−0.16	−0.27	−0.05	−0.32
Bastian et al., 2005	246	CC	Denial	T	TMMS+AES		−0.15	−0.27	−0.02	−0.30
Bastian et al., 2005	246	CC	Denial	A	MSCEIT		−0.20	−0.32	−0.08	−0.41
Zomer, 2012	300	CC	Acceptance	T	TMMS-24	R+C	0.09	−0.02	0.20	0.17
Bastian et al., 2005	246	CC	Acceptance	T	TMMS+AES		0.38	0.26	0.48	0.82
Bastian et al., 2005	246	CC	Acceptance	A	MSCEIT		0.10	−0.03	0.23	0.21
Mikolajczak et al., 2008	203	CC	Acceptance	T	TEIQue-LF		−0.06	−0.20	0.08	−0.12
Bastian et al., 2005	246	RM	Venting	T	TMMS+AES		0.05	−0.08	0.18	0.10
Bastian et al., 2005	246	RM	Venting	A	MSCEIT		0.02	−0.11	0.15	0.03
Zomer, 2012	300	RM	Venting	T	TMMS-24	R+C	−0.27	−0.37	−0.16	−0.56
Schutte et al., 2009	73	RM	suppression (ERQ)	T	EIS		−0.50	−0.66	−0.31	−1.15
Johnson and Spector, 2007	176	RM	Suppression	T	WLEIS		−0.08	−0.23	0.07	−0.16
Austin et al., 2008	247	RM	Suppression	T	TEIQue-SF		−0.45	−0.55	−0.35	−1.01
Mikolajczak et al., 2007b	124	RM	Suppression	T	TEIQue-SF		−0.31	−0.46	−0.14	−0.65
Totterdell and Holman, 2003	18	RM	Suppression	T	EIS		−0.18	−0.60	0.31	−0.36
Velasco et al., 2006	593	RM	Suppression	T	TMMS-alex F1		−0.28	−0.35	−0.21	−0.58
Kafetsios and Loumakou, 2007	475	RM	Suppression (ERQ)	T	EQ-I		−0.08	−0.20	0.04	−0.16
Cabello et al., 2013	866	RM	Suppression	T	TMMS-24	R+C	−0.13	−0.20	−0.06	−0.26
Lee and Ok, 2012	309	RM	Emotional dissonance	T	WLEIS		−0.22	−0.33	−0.11	−0.45
Rivers et al., 2013	243	RM	Aggressive behavior	A	MSCEIT		−0.25	−0.36	−0.13	−0.51
Brackett et al., 2004	330	RM	Deviant behavior	A	MSCEIT		−0.27	−0.37	−0.17	−0.56
Brackett and Mayer, 2003	207	RM	Social deviance	T	EQ-i/SREIT		−0.14	−0.27	0.00	−0.28
Shahzad et al., 2013	140	RM	Aggression	T	TEIQue-ASF		−0.31	−0.45	−0.15	−0.64
Mikolajczak et al., 2009a	490	RM	Self-harm	T	TEIQue-ASF		−0.31	−0.39	−0.23	−0.65
Karim and Shah, 2014	192	RM	Suicidal ideation	A	MSCEIT	AGS	−0.30	−0.42	−0.16	−0.63
Aradilla-Herrero et al., 2014	93	RM	Suicide risk	T	TMMS-24	R+C	−0.21	−0.40	−0.01	−0.43
Gardner et al., 2014	235	RM	Bulimic symptoms	T	MEIA		−0.22	−0.34	−0.10	−0.45
Gardner et al., 2014	235	RM	Bulimic symptoms	A	MSCEIT		−0.07	−0.20	0.06	−0.14
Gardner et al., 2014	235	RM	Binge eating	T	MEIA		−0.21	−0.33	−0.08	−0.43
Gardner et al., 2014	235	RM	Binge eating	A	MSCEIT		−0.03	−0.16	0.10	−0.06
Pettit et al., 2010	402	RM	Bulimia/Food preocupation	T	TMMS-24	R+C	−0.13	−0.23	−0.03	−0.26
Markey and Vander Wal, 2007	154	RM	Bulimic symptoms	T	EQ- i:S		−0.31	−0.45	−0.16	−0.65
Brackett et al., 2004	330	RM	Illegal drug use	A	MSCEIT		−0.11	−0.22	0.00	−0.22
Brackett et al., 2004	330	RM	Alcohol use	A	MSCEIT		−0.13	−0.24	−0.02	−0.26
Rossen and Kranzler, 2009	150	RM	Alcohol use	A	MSCEIT		−0.21	−0.35	−0.07	−0.43
Tsaousis and Nikolaou, 2005	365	RM	Alcohol units	T	TEIQ		−0.07	−0.17	0.03	−0.14
Austin et al., 2005	115	RM	Acohol consumption	T	REIS		−0.19	−0.37	−0.01	−0.38
Ghee and Johnson, 2008	214	RM	Alcohol consumption	T	EIS		−0.02	−0.15	0.11	−0.04
Riley and Schutte, 2003	141	RM	Acohol consumption	T	EIS		−0.34	−0.48	−0.2	−0.72
Brackett and Mayer, 2003	207	RM	Alcohol consumption	T	EQ-i/SREIT		−0.13	−0.26	0.01	−0.26
Trinidad and Johnson, 2002	205	RM	Alcohol consumption	A	MEIS		−0.12	−0.26	0.01	−0.25
Saklofske et al., 2007	362	RM	Alcohol consumption	T	EIS		−0.05	−0.15	0.05	−0.10
Brackett and Mayer, 2003	207	RM	Illegal drug user scale	T	EQ-i/SREIT		−0.14	−0.27	−0.003	−0.28
Riley and Schutte, 2003	141	RM	Drug abuse	T	EIS		−0.42	−0.55	−0.29	−0.92
Limonero et al., 2006	133	RM	Canabis smoking	T	TMMS	R+C	−0.01	−0.19	0.16	−0.02
Bastian et al., 2005	246	RM	Substance use	T	TMMS+AES		−0.05	−0.18	0.08	−0.10
Bastian et al., 2005	246	RM	Substance use	A	MSCEIT		−0.02	−0.14	0.11	−0.03
Rivers et al., 2013	243	RM	Substance abuse	A	MSCEIT		−0.18	−0.31	−0.06	−0.36
Schutte et al., 2011	100	RM	Alcohol problems	A	MSCEIT		−0.30	−0.46	−0.11	−0.63
Schutte et al., 2011	100	RM	Alcohol problems	T	AES		−0.27	−0.45	−0.08	−0.56
Zomer, 2012	300	RM	Drugs	T	TMMS-24	R+C	−0.11	−0.22	0.00	0.21
Monaci et al., 2013	198	RM	Alcohol use	T	SEIS		−0.05	−0.19	0.09	−0.10
Solanki and Lane, 2010	315	RM	Exercise mood regulating	T	EIS		0.45	0.35	0.53	1.01

*SS, Situation Selection; SM, Situation Modification; AD, Attentional Deployment; CC, Cognitive Change; RM, Response Modulation; T/A, Trait or Ability EI measure: T, trait EI measure, A, ability EI measure; AGS, Aggregated Global Score; R+C, Repair + Clarity; AES, Assessing Emotions Scale; EIS, Emotional Intelligence Scale; EQ-i/YV/S, Emotional Quotient Inventory/Young Version/Short; WLEIS, The Wong and Law Emotional Intelligence Scale; SREIT, Self Report Emotional Intelligence Test; TMMS-24, Trait Meta Mood Scale; TEIQ, The Traits Emotional Intelligence Questionnaire; TEIQue/SF/LF/ASF, Trait Emotional Intelligence Questionnaire/ Short-Form/Long-Form/Adolescent Short-Form; MSCEIT, The Mayer-Salovey-Caruso Emotional Intelligence Test; SEIS, Schutte Emotional Intelligence Scale; SREIS, Self-Reported Emotional Intelligence Scale; EQ-Index, Emotional Quotient Index; WEIP6, Workgroup Emotional Intelligence Profile-Version 6; EII-R, Emotional Intelligence Inventory Revised; MEIS, Multifactoral Emotional Intelligence Scale; EJI, Emotional Judgment Inventory; SSRI, Schutte Self-Report Inventory; MEIA, Multidimensional Emotional Intelligence Assessment; REIS, Revised Emotional Intelligence Scale; SEI, Survey of Emotional Intelligence. JET, Judgment of Emotions Test; IJI, Interpersonal Judgment Inventory; STEU, Situational Test of Emotional Understanding*.

^*^The forecast accuracy indices were calculated such that higher numbers indicate poorer accuracy.

a *The core self-evaluation construct is a fundamental part of self-evaluated values, efficacy and abilities. It includes self-esteem, self-efficacy, internal locus of control and absence of pessimism*.

#### Statistical analyses

Each study provided us with a measure of association between EI and (at least) one ER strategy. The effect size of EI on this strategy was expressed in Pearson's *r* in the papers and we therefore computed all the individual confidence intervals around *r*. In order to compute these confidence intervals, Fisher's *r*-to-z' transformation was used. First, *r* was converted to *z*'. Second, the confidence intervals were computed in terms of *z*'. Taking into account that the *z* for a 95% confidence interval (*Z*_0.95_) is 1.96 and that the standard error is calculated by the formula 1/√N−3, the lower limit confidence interval was computed by the next formula: lower limit *z*' = *z*' − (1.96) (standard error). Regarding the upper limit confidence interval, the used formula was this: upper limit *z*' = z' + (1.96)(standard error). Finally, these confidence intervals (computed in terms of *z*') were then back-transformed to *r* getting the confidence intervals in terms of *r*. As readers are usually more familiar with effect sizes expressed in Cohen's “*d*” (whose interpretation is facilitated by Cohen's norms), each effect size “*r*” was then converted into “*d*.” The formula for converting between *r* and *d* were taken from Rosenthal (1994, p. 239). In a third step, we aggregated the relevant individual effect sizes to obtain the aggregate effect size of EI on each ER family. When several effects of a given family came from the same study, they were first aggregated and this aggregated effect size was then aggregated with the effect sizes of the other studies. In order to give more weight to the effect sizes coming from studies with larger samples, we used the methods suggested by Lipsy and Wilson (2000): The “Inverse Variance Weight” was used to compute the standard error and the weight of each effect. The aggregated effect size consists of the sum of the weight and the effect size multiplication, divided by the sum of the weights. Finally, confidence intervals were calculated by taking the effect size (Cohen's *d*) and the standard error into account.

## Results

We review below the findings of the studies that have investigated the relationship between EI and ER. Each family is presented in turn. Each section begins with results relative to trait EI which form the majority of the studies analyzed. The sections then turn to results regarding ability EI when these are available. The effect sizes (with confidence interval) of EI in each study are reported in Table 1. The aggregated effect size (with confidence interval) of EI on each ER family is reported in Table 2 for trait EI and Table 3 for ability EI.

**Table 2:** **Linking emotional intelligence (trait) to the use of emotion regulation strategies**.

**ER family**	**ER strategy**	**Number of studies**	**Total *N***	**Dir. of effect**	**Effect-size (*d*)**	**95% Confidence Interval Interval around *d***
Situation selection	Forecast accuracy^*^	1	84	−	−0.18	[−0.40; 0.04]
	Forecast accuracy	2	162	+	0.51	[0.35; 0.66]
	Situation selection	1	73	+	0.63	[0.39; 0.86]
	Daily hassles	2	435	−	−0.30	[−0.39; −0.20]
	Time to relax	1	365	+	0.95	[0.85; 1.06]
	Avoidant-coping	10	3136	−	−0.27	[ −0.31; −0.24]
	Perseverance	2	75	+	1.02	[0.78; 1.25]
	Behavioral disengagement	1	246	−	−0.49	[−0.58; −0.40]
Situation modification	Situation modification	1	73	+	0.41	[0.17; 0.64]
	Problem solving	24	6516	+	0.92	[0.90; 0.94]
	Problem solving (negative)	1	492	−	−1.06	[−1.19; −0.93]
	Social support search	8	2437	+	0.38	[0.34; 0.40]
	Constructive conflict resolution	5	1268	+	0.56	[0.50; 0.61]
	Conflict resolution (avoid)	4	948	−	−0.04	[−0.11; 0.02]
	Conflict resolution (dominate)	3	809	+	0.27	[0.20; 0.34]
	Restraint	1	246	+	0.16	[0.03; 0.29]
Attentional deployment	Attention deployment	1	73	+	0.82	[0.59; 1.06]
	Focus on positive	1	18	+	1.04	[0.53; 1.54]
	Rumination	6	1989	−	−0.43	[−0.47; −0.38]
	Distraction	5	1210	+	0.26	[0.21; 0.32]
	Mindfulness	6	1646	+	0.85	[0.80; 0.90]
Cognitive Change	Challenge appraisal	2	85	+	0.35	[0.13; 0.57]
	Threat appraisal	1	70	−	−0.90	[−1.14; −0.66]
	Self-efficacy	20	3727	+	1.08	[1.05; 1.11]
	Reappraisal	10	2902	+	0.61	[0.58; 0.65]
	Adaptive humour	3	1584	+	0.72	[0.67; 0.76]
	Maladaptive humour	1	1038	−	−0.55	[−0.61; −0.49]
	Denial	3	798	−	−0.27	[−0.34; −0.20]
	Acceptance	3	749	+	0.31	[0.23; 0.38]
	Cognitive change	1	73	+	0.54	[0.30; 0.77]
Response modulation	Exercise as a mood regulating strategy	1	315	+	1.01	[0.90; 1.12]
	Venting	2	546	−	−0.26	[−0.35; −0.18]
	Suppression	9	2881	−	−0.43	[−0.47; −0.39]
	Aggressivity	2	347	−	−0.43	[−0.53; −0.33]
	Self−harm	2	583	−	−0.62	[−0.70; −0.53]
	Substance use	13	2729	−	−0.25	[−0.29; −0.21]
	Bullimia/Food preocupation	4	1026	−	−0.40	[−0.46; −0.34]

* *The forecast accuracy indices were calculated such that higher numbers indicate poorer accuracy*.

**Table 3:** **Linking emotional intelligence (ability) to the use of emotion regulation strategies**.

**ER family**	**ER strategy**	**Number of studies**	**Total *N***	**Dir. of effect**	**Effect-size (*d*)**	**95% Confidence Interval around *d***
Situation selection	Forecast accuracy^*^	1	84	–	−0.45	[−0.67; −0.23]
	Forecast accuracy	2	162	+	0.81	[0.65; 0.97]
	Avoidant coping	1	159	–	−0.43	[−0.59; −0.27]
	Behavioral disengagement	1	246	–	−0.32	[−0.44; −0.19]
Situation modification	Problem solving	3	628	+	0.23	[0.15; 0.31]
	Problem solving (negative)	1	246	–	−0.08	[−0.21; 0.05]
	Social support seeking	2	469	+	0.10	[0.01; 0.19]
	Conflict resolution	1	200	+	0.49	[0.35; 0.63]
	Conflict resolution (avoid)	1	200	–	−0.85	[−0.99; −0.71]
	Restraint	1	246	+	0.15	[0.02; 0.28]
Attentional deployment	Rumination	1	157	–	−0.98	[−1.14; −0.82]
	Mental disengagement	1	246	–	−0.10	[−0.23; 0.03]
Cognitive change	Self-efficacy	3	564	+	0.47	[0.33; 0.61]
	Positive interpretation	1	246	+	0.21	[0.08; 0.34]
	Humour	1	246	+	0.08	[−0.05; 0.21]
	Denial	1	246	–	−0.41	[−0.54; −0.28]
	Acceptance	1	246	+	0.21	[0.08; 0.34]
Response modulation	Venting	1	246	+	0.27	[0.14; 0.40]
	Aggressive behavior	2	573	–	−0.54	[−0.62; −0.46]
	Self-harm	1	192	–	−0.63	[−0.77; −0.49]
	Substance use	7	1604	–	−0.27	[−0.32; −0.22]
	Bulimia/Food preocupation	2	470	–	−0.10	[−0.19; −0.01]

* *The forecast accuracy indices were calculated such that higher numbers indicate poorer accuracy*.

### EI and situation selection

As we previously mentioned, selecting situations depending on the expected emotional impact requires being able to accurately forecast how one is likely to feel in different situations, and then use that information to confront situations that are likely to bring long-term benefits, while avoiding the others. Accordingly, we hypothesized that high EI people would make more accurate affective forecasts comparing to their low EI peers. We also hypothesized that high EI individuals would do their best to prevent negative situations to occur (which should result in less daily hassles) but that they not avoid negative/stressful situations if they were to bring long-term benefits. In the latter case, high EI people would on the contrary struggle to deal with the situation rather than giving up their goal.

Empirical evidence suggests that trait EI increases affective forecasting accuracy (Hoerger et al., 2012). It must nonetheless be noted that Dunn et al. (2007) found only a non-significant trend in a smaller sample. Schutte et al. (2009) have shown that people with high trait EI use that knowledge to select situations more effectively (i.e., spend time in situations that boost wanted emotions or prevent unwanted emotions, seek out situations that inspire positive emotions or avoid those that arouse negative emotions). This may explain why two studies (Ciarrochi et al., 2002; Day et al., 2005) found negative correlations between trait EI and the self-reported frequency of various daily hassles such as marital, professional, health, financial and relationship hassles. It is also congruent with the findings that high trait EI people take more time to relax and wind down (Tsaousis and Nikolaou, 2005).

It is noteworthy that high EI individuals do not simply avoid all situations that could cause them negative emotions. As shown in Table 2, they actually report using *less* avoidant coping strategies than their peers (Gerits et al., 2004; Petrides et al., 2007a,b; Mikolajczak et al., 2009a, Studies 1 and 2; Rogers et al., 2006; Velasco et al., 2006; Shah and Thingujam, 2008; Kim and Agrusa, 2011; although see Monaci et al., 2013 for insignificant results). This is probably because many situations that cause negative emotions in the short-term have long-term benefits (academic exams for instance). Experimental and field studies support the idea that high trait EI individuals tend to struggle (confront) rather than give up (escape/avoid) in the face of adversity if the confrontation is likely to bring about more substantial long-term benefits. For example, an experimental study by Schutte et al. (2001b) showed that participants who scored higher in trait emotional intelligence solved more problems after encountering a very difficult and frustrating set of problems. This held true even when initial performance was held constant. In the same vein, a field study by Petrides et al. (2006a) showed that trait EI was positively related to the length of musical training among music school students, suggesting that high trait EI individuals do not let themselves be discouraged by the obstacles that stand in their way. Consistent with these results, Bastian et al. (2005)^7^ found that EI was associated to “behavioral disengagement” as people with high EI made an effort to deal with the stressor, and did not give up the attempt to attain goals with which the stressor was interfering.

The studies that investigated ability EI in relation to situation selection strategies show that people with high ability EI make more accurate affective forecasts (Dunn et al., 2007; Hoerger et al., 2012), use less avoidant coping strategies (MacCann et al., 2011) and strive more to attain their goals (Bastian et al., 2005)^8^.

### EI and situation modification

We hypothesized that high EI individuals would take steps to modify a disadvantageous situation. We also expect them to make use of both their social skills and their aptitude to express emotions to prompt situation modification when this requires the intervention of a third party. Therefore, we predicted that high EI individuals would use constructive conflict resolutions rather than avoidant ones. Finally, we hypothesized a positive relationship between EI and restraint (i.e., waiting for the appropriate moment to act, avoiding to act prematurely).

Empirical evidence indicates that when confronted with a negative situation, high trait EI individuals are more likely to modify the situation (Schutte et al., 2009) and take action to change things than their low trait EI counterparts (Salovey et al., 2002 (Study 3); Rahim and Minors, 2003; Gerits et al., 2004; Bastian et al., 2005,^9^; Goldenberg et al., 2006; Rogers et al., 2006; Velasco et al., 2006; Petrides et al., 2007a,b; Saklofske et al., 2007, 2012; Almran and Punamaki, 2008; Kluemper, 2008; Mikolajczak et al., 2008, 2009a; Shah and Thingujam, 2008; Austin et al., 2010; Noorbakhsh et al., 2010; Kim and Agrusa, 2011; Moradi et al., 2011; Zomer, 2012; Monaci et al., 2013; Tsarenko and Strizhakova, 2013; although see Montes-Berges and Augusto, 2007 for null results). The relationship between trait EI and the restraint coping strategy does not reach significance though. Namely, people with high trait EI are not significantly more able than people with low trait EI to await the appropriate opportunity before taking action (that is, holding one-self back and not acting prematurely) (Bastian et al., 2005)^10^.

Direct problem solving is not the only strategy used by high trait EI individuals when it comes to modifying a situation. As expected, these individuals also make use of indirect modification strategies. First, high trait EI individuals report being more willing to seek help from friends, family, and health professionals in case of problems (Ciarrochi and Deane, 2001; Gerits et al., 2004; Bastian et al., 2005,^11^; Goldenberg et al., 2006; Velasco et al., 2006; Zomer, 2012; Monaci et al., 2013; although see Shah and Thingujam, 2008, for insignificant results). Note that obtaining adequate support may also be easier for them because EI has been associated with increased perceived quantity and quality of social support (e.g., Austin et al., 2005; Mikolajczak et al., 2007a).

Second, high EI people are, by definition, more able and inclined to freely express both positive and negative emotions which, according to Petrides and Furnham's model (2003), translates into higher assertiveness. Thus, instead of fuming at the cigarette smoke of their neighbor at table, high EI individuals would typically ask to have the cigarette put out. However, studies are needed to further test this claim. Third, EI seems to be related to more constructive conflict resolution strategies, although the pattern of results is not entirely clear (Jordan and Troth, 2002, 2004; Salami, 2010b).

Regarding ability EI, the results obtained are consistent with those that use trait measures. Higher ability EI is associated with greater use of problem-focused coping (Goldenberg et al., 2006; MacCann et al., 2011; but see Bastian et al., 2005,^12^ for non-significant results), although there is no significant relationship between an individual's ability to restrain him or herself (wait for the appropriate moment to act and avoid acting prematurely) and EI (Bastian et al., 2005)^13^. Ability EI also relates to more social support seeking, although effect sizes are nearly null (see Bastian et al., 2005,^14^; Goldenberg et al., 2006).

### EI and attentional deployment

Given that high EI people are characterized by greater positive trait affectivity, we hypothesized that they would pay greater attention to positive stimuli/events and ruminate less about negative events. It has indeed been shown that rumination on sad or angry events increases the duration and intensity of negative emotions (Morrow and Nolen-Hoeksema, 1990; Bushman, 2002). Unlike rumination, it is not clear how EI should be related to distraction.

Empirical evidence indicates that high trait EI people rely heavily on attentional deployment techniques to regulate their emotions. First, people with higher trait EI report more mindful attention awareness and pay greater, non-judgmental attention to the present moment (Brown and Ryan, 2003; Baer et al., 2004 (Study 4); Kokinda, 2010; Schutte and Malouff, 2011; Charoensukmongkol, 2014; Wang and Kong, 2014). Consistent with this finding, they report ruminating less about negative/stressful events than their lower EI counterparts (Salovey et al., 2002 (Study 3); Petrides et al., 2007a,b; Ramos et al., 2007; Salguero et al., 2013, although see Mikolajczak et al., 2008 for non-significant results). Schutte et al. (2009) show that higher trait EI people report paying greater attention to things that either help arouse the emotions they desire or prevent them from experiencing emotions they seek to avoid. Similarly, Totterdell and Holman (2003) have shown that high trait EI individuals focus more on positive things than people with low trait EI. These individuals also report that they make more use of distraction to regulate their emotions than their peers (Salovey et al., 2002 (Study 3); Bastian et al., 2005,^15^; Mikolajczak et al., 2008; Saklofske et al., 2012; although see Austin et al., 2010 for non-significant results).

Results concerning ability EI seem to be less consistent. While Lanciano et al. (2012) found that high ability EI individuals ruminate less than their equivalents, Bastian et al. (2005)^16^ showed no significant relationship between ability EI and mental disengagement. Future studies need to examine further the relationship between ability EI and attentional deployment strategies.

### EI and cognitive change

We predicted a positive relationship with challenge appraisal because high EI individuals' positive dispositions should lead them to consider not only the potential losses but also potential gains inherent to a situation. Likewise, we predicted a positive link between EI and self-efficacy because high EI individuals' previous successful attempts to regulate their emotions should enhance their confidence to deal with negative events. We also predicted a positive relationship with strategies such as reappraisal and adaptive humor which are useful when the situation cannot be prevented, directly modified, or cognitively avoided (e.g., an academic exam). Finally, we predicted a positive relationship between trait EI and acceptance, but only for problems that could not be modified or reappraised (e.g., being diagnosed with terminal cancer).

Empirical studies show that trait EI is related to how one views a given situation. When asked in January (exam period) how threatening/challenging they appraised the exam session, high EI freshmen judged it as much less threatening than their low EI peers (Mikolajczak et al., 2006). These findings were replicated twice in a laboratory setting. Mikolajczak and Luminet (2008) found that high EI individuals tended to appraise an upcoming arithmetic task as a challenge whereas low trait EI individuals tended to appraise it as a threat.

In addition to influencing how people perceive a given situation, EI also affects how one views one's ability to manage the demands the situation poses (naturally, the two are related). In the above-mentioned study (Mikolajczak et al., 2006), when asked at the beginning of the academic year about their self-efficacy to manage the January exam session, high EI freshmen reported higher self-efficacy than low EI individuals. This was all the more interesting as there was no correlation between trait EI and general cognitive ability (IQ; e.g., Mikolajczak et al., 2007a). In order to rule out potentially confounding variables such as prior knowledge of the subjects or social support, the study was replicated within a laboratory setting (two studies). High trait EI people reported greater self-efficacy to deal with the tasks (analysis of the psychological profile of a movie character in Study 1; an arithmetic task in Study 2) than their low trait EI peers (Mikolajczak and Luminet, 2008). These results are consistent with many others that reveal a relationship between trait EI and self-efficacy (see Adeyemo, 2007 for self-efficacy to pass university exams; Brown et al., 2003 for career decision-making self-efficacy; Animasahun, 2008 for generalized self-efficacy; Chan, 2004 for general self-efficacy; Charoensukmongkol, 2014 for general self-efficacy; Di Fabio and Palazzeschi, 2008 for self-efficacy of teachers to deal with their classroom; Di Fabio and Saklofske, 2014a,b for core self-evaluation and career decision self-efficacy; Durán et al. (2006) for general self-efficacy and self-efficacy to succeed at university; Kaur et al., 2006 for self-efficacy to restrain from gambling; Kirk et al., 2008 for emotional self-efficacy; Kluemper, 2008 for core self-evaluation; Martin et al., 2004 for self-efficacy in counseling; Mouton et al., 2013 for self-efficacy among physical education teachers; Tsarenko and Strizhakova, 2013 for consumer self-efficacy; and Villanueva and Sanchez, 2007 for self-efficacy to perform a laboratory task and to coach and lead followers to perform a laboratory task; although see Salami, 2010a for a non-significant effect of trait EI on general self-efficacy).

When the initial^17^ appraisal of a situation and of one's ability to manage it is not sufficient to achieve the desired emotional state, individuals with higher trait EI report being more prone to change the way they think in order to feel or prevent particular emotions (Schutte et al., 2009). In line with this, high trait EI people report greater use of reappraisal strategies than low EI people (Totterdell and Holman, 2003; Bastian et al., 2005,^18^; Velasco et al., 2006; Mikolajczak et al., 2008; Shah and Thingujam, 2008; Schutte et al., 2009; Moradi et al., 2011; Cabello et al., 2013 although see Kafetsios and Loumakou, 2007, for unsignificant correlations). It must be noted that these results were not replicated by Coumans (2005) who asked participants about the utilization of several coping strategies after a forced failure in a cognitive task. She found that high EI individuals were not more likely than their low EI peers to report using positive reappraisal strategies such as self-esteem rebuilding, putting things into perspective, trying to accept the situation as a part of life, or looking for the silver lining. Future studies are clearly needed to delineate the moderators and boundary conditions of this effect. It is worth noting that the tendency of high EI people to change the way they think in order to modify their feelings does not go as far as denying the problem. Indeed, people with high trait EI report using less denial strategies (Bastian et al., 2005,^19^; Zomer, 2012; Tsarenko and Strizhakova, 2013). Besides positive reappraisal, it seems that higher trait EI is associated with greater use of humor (Bastian et al., 2005,^20^; Greven et al., 2008; although see Zomer, 2012 for non-significant results) and less maladaptive humor (Greven et al., 2008). Finally, it must be noted that to date, how trait EI relates to acceptance remains unclear. Although Bastian et al. (2005)^21^ found that high EI individuals reported accepting life events more easily, two other studies found no significant correlation between high EI and acceptance (Mikolajczak et al., 2008; Zomer, 2012).

As regards ability EI, these studies are consistent with the results obtained using trait measures in various ways. First, there is a positive relationship between high ability EI and self-efficacy (Kirk et al., 2008; Di Fabio and Saklofske, 2014a,b). Second, people with a high ability EI score report using less denial compared to their peers (Bastian et al., 2005)^22^. However, the relationship between ability EI and positive interpretation, acceptance and humor is not significant (Bastian et al., 2005)^23^.

### EI and response modulation

Because high EI people are expected to have achieved their regulatory goal (namely, the desired emotional state) through the use of the four families of strategies reviewed above, we predicted a negative relationship between trait EI and response-modulation strategies.

As predicted, there is a negative relationship between EI and most response modulation strategies, or at least those strategies whose relationship with EI has been investigated. The only exception concerns “exercise as a mood-regulation strategy,” which shows a positive correlation with trait EI (Solanki and Lane, 2010). To the best of our knowledge, no study has yet examined the relationship between EI and the social sharing of emotions. We would nevertheless expect a negative relationship between EI and the number of times a given emotional episode is shared. Indeed, the more intense the emotion, the greater the extent of sharing (Rimé, 2009). As high scoring EI people have more (and more functional) strategies at their disposal to regulate their emotions, we would expect that they would have less need for emotion sharing. Moreover, we would expect them to better choose the recipient and moment for sharing; they would thus feel better listened to, thereby reducing the need for further sharing.

While no study on trait EI and social sharing exists, two studies have examined the relationship between trait EI and venting (i.e., expressing negative feelings, usually one's anger). Consistent with our expectations, one study found that higher trait EI participants were less likely to vent (Zomer, 2012). However, the other study found no relationship between trait EI and venting (Bastian et al., 2005)^24^.

A number of studies have analyzed EI and other response-modulation strategies such as expressive suppression, aggression, and substance abuse. As far as expressive suppression is concerned, high trait EI people report suppressing their emotions less (Totterdell and Holman, 2003; Velasco et al., 2006; Johnson and Spector, 2007; Mikolajczak et al., 2007b; Austin et al., 2008; Schutte et al., 2009; Lee and Ok, 2012; Cabello et al., 2013; although see Kafetsios and Loumakou, 2007 for null findings), probably because they have already achieved their desired emotional state and/or because they value genuine emotion expression. Two pieces of evidence support the hypothesis that high trait EI individuals' lesser use of suppression is attributable to a lesser need rather than a lesser capacity. First, although high trait EI people report making less use of suppression, they do not report feeling less capable than their low EI peers to use it when required (Mikolajczak et al., unpublished data). Second, in a study of service workers' emotional labor^25^, high trait EI employees reported experiencing less emotional dissonance (i.e., dissonance between their inner feelings and the feelings required by organizational display rules) than low EI people. High EI individuals thus needed to perform reappraisal and suppression less frequently than low EI individuals (Mikolajczak et al., 2007b).

As expected, trait EI was also negatively linked to both verbal and physical aggression (Brackett and Mayer, 2003; Shahzad et al., 2013), suggesting that high EI people can defuse their anger and frustration through other means. We must draw attention to the fact that people with high trait EI do not seem to turn their aggressiveness against themselves. There is indeed a negative correlation between trait EI and self-harm (Mikolajczak et al., 2009a), including suicidal attempts (Aradilla-Herrero et al., 2014). High EI people do not therefore need to resort to self-harm to reduce unwanted emotions.

Finally, trait EI is negatively associated with the consumption of alcohol (Brackett and Mayer, 2003; Austin et al., 2005; Schutte et al., 2011; although see Tsaousis and Nikolaou, 2005; Saklofske et al., 2007; Ghee and Johnson, 2008; Monaci et al., 2013 for null results), suggesting that high trait EI people do not need alcohol to anesthetize their feelings. Moreover, they do not seem to (ab)use food to regulate their emotions as there is a negative relationship between trait EI and both Body-Mass Index (Swami et al., 2010), binge eating (Gardner et al., 2014), and bulimia (Markey and Vander Wal, 2007; Pettit et al., 2010; Gardner et al., 2014). It must be noted that the findings on the relationship between trait EI and the use of cannabis and other drugs are inconsistent. While some studies have found the expected negative relationship (Brackett and Mayer, 2003; Riley and Schutte, 2003; Tsaousis and Nikolaou, 2005; Limonero et al., 2006; Rivers et al., 2013), other studies have found non-significant correlations (Bastian et al., 2005,^26^; Limonero et al., 2006; Saklofske et al., 2007; Zomer, 2012).

Regarding ability EI, results are more or less consistent with studies that have used trait measures. There is no significant association between ability EI and venting emotions (Bastian et al., 2005)^27^. Subjects with high EI ability display less aggressive behavior (Brackett et al., 2004; Rivers et al., 2013), less suicidal ideation, fewer suicidal attempts (Karim and Shah, 2014) and are less likely to use drugs (Trinidad and Johnson, 2002; Brackett and Mayer, 2003; Brackett et al., 2004; Rossen and Kranzler, 2009; Schutte et al., 2011; Rivers et al., 2013; although see Bastian et al., 2005,^28^ for null results). The correlation between ability EI and bulimic symptoms and binge eating is non-significant (Gardner et al., 2014).

## Toward a process conception of EI

Our review suggests that different levels of EI are associated with different patterns of emotion regulation use. In particular, high EI individuals shape their emotion trajectory at the earliest possible point. This hardly implies that they only expose themselves to positive situations. Indeed, high EI individuals confront rather than avoid negative situations if this may lead to more considerable or sustainable long-term benefits (e.g., university exams). However, when caught in a negative situation, high EI individuals use all possible means to modify the situation and alter its emotional impact. If possible, they directly attempt to modify the situation (e.g., study sufficiently in order to make the examination less stressful). They also seek and make use of their social support (e.g., call parents for reassurance or ask a friend to explain to them a poorly understood subject). If high EI individuals cannot modify a situation, they are likely to try and transform it indirectly. For instance, they can achieve this by expressing their emotions (e.g., telling a professor that they feel stressed in order to make him or her more compassionate and sensitive). If there is no way to modify the situation (e.g., if they anticipate that telling the professor how stressed they are would make him or her react negatively), high EI individuals distract themselves instead of ruminating about it. In addition, they strive to remain confident about their ability to cope with the situation. They also try to change the way they perceive the situation in order to change how they feel about it (e.g., thinking that the exam is nothing more than a test of knowledge and that the stakes are ridiculously low compared to people risking their lives during the war). Because high EI individuals are able to alter the trajectory of their emotional experience early on, they do not need to suppress their behavioral emotional manifestations (e.g., control one's hand or voice trembling) or anesthetize their feelings using alcohol, food, or self-harm.

Because different forms of emotion regulation have divergent outcomes, the *consequences* of a potentially emotion-eliciting event should be markedly different for high and low EI people. Since high levels of emotional intelligence are associated with strategies traditionally viewed as adaptive (i.e., generally associated with decreased subjective experience and peripheral physiological arousal), high EI individuals are expected to display less emotional reactivity in response to negative emotion-eliciting situations.

One approach to examining this issue is using stressful laboratory tasks. These situations typically involve a task (e.g., public speaking, arithmetic) to be performed under stressful conditions (e.g., the presence of an evaluative audience, time pressure). This paradigm is of interest as it induces negative emotions but, at the same time, creates implicit pressure toward emotion regulation because performance is impaired by excessive emotional reactivity. Using a similar paradigm on several occasions, Mikolajczak and colleagues repeatedly found that high EI individuals displayed lesser emotional reactivity than low EI individuals. Specifically, compared to low EI participants, high EI participants displayed less mood deterioration (Mikolajczak et al., 2007c, 2009b), less emotional intensity, action tendencies, bodily sensations (Mikolajczak et al., 2007a), and less cortisol secretion (Mikolajczak et al., 2007c) in response to the stressor. Consistent with these findings, other researchers have found that high EI people display less regret after failed negotiations than their low EI peers (Sevdalis et al., 2007). This higher ego-resiliency has also been revealed by Schutte et al. (2002). Their study found that following a negative mood-induction using the Velten method (sentences meant to provoke a drop in self-esteem), there was a lesser decrease in positive affect and self-esteem among higher trait EI individuals than among lower trait EI individuals.

An important question raised by these studies is whether high trait EI individuals regulate their emotions better than their lower counterparts, or whether they are merely less reactive to affective stimuli/situations in general. The results of a second type of studies provide preliminary evidence that trait EI is not associated with uniform lower sensitivity to affective cues. Rather, it appears to be linked to a relatively flexible functioning, promoting either increased or decreased sensitivity to affect-laden stimuli, depending on the context.

The studies reviewed above examined sensitivity to *stressful* situations, which, by definition, threaten one's goals and integrity, and therefore call for immediate mood regulation. By contrast, a second kind of studies did not involve any implicit or explicit pressure toward emotion regulation. These studies simply presented research participants with neutral, negative (e.g., sad, anger-eliciting) and/or cheerful (e.g., amusing) video clips. Contrary to “stress-inducing” studies, the second type of studies either found no clear moderating effect of EI on immediate reactivity to the videos (Ciarrochi et al., 2001) or found higher EI scores associated with *increased* reactivity (Petrides and Furnham, 2003). These results suggest that EI is actually sensitive to affective cues and leaves room for emotions to emerge. Nevertheless, these studies also show that high EI individuals are more likely to implement efficient regulation strategies after the negative videos than their low EI peers. Specifically, Ciarrochi et al. (2001) found a significant moderating effect of EI on the valence of stories composed following the clips, suggesting that high EI participants are more able to generate positive stories in order to maintain a positive mood/repair a negative mood than low EI people. Likewise, Petrides and Furnham (2003) found that high EI individuals were more able to use subsequent cheerful videos to repair their mood than low EI people.

Taken together, these findings suggest that trait EI is associated with differences in emotion regulation rather than with differences in reactivity to emotion-laden stimuli (see also Sevdalis et al., 2007). Early EI theorists proposed that the salient feature of “emotionally intelligent regulation” was its flexibility: EI could not be characterized by a constant regulation resulting in the absence of emotion. On the contrary, emotionally intelligent individuals could be quite open to emotions, take advantage of the information they convey, and efficiently regulate this information when it became redundant or inappropriate (Mayer and Salovey, 1997). The findings reviewed above are clearly in line with this conceptualization.

## Future directions in the study of emotional intelligence and emotion regulation

In the previous section, we analyzed how EI relates to emotion regulatory processes and outcomes. It is noteworthy that although many processes were reviewed, not all emotion-regulation processes were mentioned because some processes were not investigated in relation to EI. Some of the processes awaiting investigation in relation to EI include those pertaining to interpersonal emotion regulation (see, e.g., Rimé, 2007 for the benefits of the social sharing of emotions) or to the processing of emotional information (see, e.g., Philippot et al., 2003 for the benefits of specificity in emotional information processing). In addition to highlighting the need to further address these specific issues, the present review also opens up broad avenues for future research. In the following sections, we will present several promising directions, each of which is likely to broaden and extend our understanding of the relationship between EI and emotion regulation.

### Up-regulation vs. down-regulation

The studies reviewed above have focused on the down-regulation of negative emotions, by far the most common target of emotion regulation efforts (Gross et al., 2006). However, although this is a reasonable starting point, much remains to be done. As emphasized elsewhere (Gross, 2014), emotion regulation can occur anywhere in the 2 × 2 matrix formed by crossing negative and positive emotions with up- and down-regulation. Consequently, people not only try to decrease negative emotions (Gross et al., 2006), they also try to increase positive ones (Quoidbach et al., 2010, 2015). On some occasions, they also strategically increase negative emotions (e.g., anger when collecting debts, Sutton, 1991; anger before engaging in a confrontational task, Tamir et al., 2008; worry and fear when anticipating a threatening task, Tamir et al., 2007), or decrease positive emotions (e.g., amusement during a serious meeting; Gruber et al., 2011).

We believe that research on individual differences in emotion regulation would greatly benefit from integrating research all four facets of the emotion regulation goals matrix. For instance, are high EI individuals are as efficient in *up*regulating negative emotions as in *down*regulating them? The up- and down-regulation of *positive* emotions must also be examined. This kind of research is especially warranted as it is not obvious, for instance, that EI will be conductive of efficient down-regulation of *positive* emotions. It may well be the case that high trait EI individuals experience difficulties in down-regulating positive emotions and up-regulating negative emotions. Studies that delve deeper into these questions are crucial as this issue has both theoretical and practical implications.

At the theoretical level, it is important to know whether an individual can perform at above average levels in each of the 2 × 2 ER matrix cells, or whether the price to pay for above average down-regulation of negative emotions/up-regulation of positive emotions is below average up-regulation of negative emotions/down-regulation of positive emotions. If the latter is true, EI definitions and theories should be amended accordingly. At the practical level, if measures of trait EI capture people who are skilled in the down-regulation of negative emotions/up-regulation of positive emotions but whose down-regulation of positive emotions/up-regulation of negative emotions is impaired, then those scoring high on EI measures would not be suitable candidates for positions such as bill collectors, funeral directors and so on. Research is also needed to understand how EI affects composite instances of emotion regulation. Indeed, individuals sometimes try to regulate multiple and conflicting emotions such as simultaneous feelings of pride about one's achievement and sadness/concern about the failure of a friend, or both joy and guilt when one indulges in some forbidden pleasure.

## Automatic vs. effortful emotion regulation

Contemporary dual-process models contrast automatic (also called implicit or non-conscious) processes with deliberate (also called explicit, conscious or controlled) processes (e.g., Strack and Deutsch, 2004). Whereas deliberate processes require attentional resources, are volitional, conscious, and goal-driven, automatic processes require neither attention nor intention, occur outside of awareness, and are stimulus driven.

The notion that relatively high-level self-regulatory processes such as emotion regulation can be performed automatically may seem counterintuitive (Bargh, 2004). However, there is ample evidence that the full sequence of goal pursuit—from goal setting to the completion of the goal—can proceed outside of conscious awareness. For instance, Bargh et al. (2001) have shown that priming the goal of achievement led research participants to outperform a control group on a variety of tasks, and subliminal priming of cooperation led participants to make a greater number of cooperative responses in a “commons dilemma” situation. Interestingly, participants behaved in line with these goals without knowing *why* or even *that* they were acting this way. Even more remarkable, the outcomes were the same when the goal was primed and operated outside of awareness as when the goal was explicitly stated in the task instructions (see Fitzsimons and Bargh, 2004 for a review).

The few studies that have investigated whether emotion regulation can also operate automatically have provided preliminary support for this idea. For instance, Mauss et al. (2007) have shown that participants primed with emotion control reported less anger than participants primed with emotion expression following an anger-induction manipulation.

EI research to date has not sought to determine whether emotion regulation processes displayed by high vs. low EI people are automatic or effortful. One may be inclined to conclude that high EI is associated with largely automatic regulation processes. There are two main arguments in favor of this view. First, as suggested by Bargh and Williams (2007), insofar as an individual performs emotion regulation routinely and in a relatively consistent manner, this process must follow the principles of skills acquisition and become progressively more automatic. Second, and according to the same authors, automatic emotion regulation processes are much more consistent and reliable than conscious processes and have the advantage of operating effectively even in the presence of cognitive load (because automatic processes do not require attentional resources). Thus, insofar as high EI individuals are able to regulate their emotions while under a cognitive load (e.g., preparing a public speech in Mikolajczak et al., 2007c, or reading sentences in Schutte et al., 2002), one might reasonably assume that emotion regulation processes occur somewhat automatically.

But things may not be quite so simple. As stated above, automatic processes occur outside of awareness and are thus stimulus-driven, which in the case of emotion regulation means “driven by emotion.” If regulatory processes were automatically initiated in response to emotions, this would leave little room for flexibility. Yet we earlier demonstrated that high EI individuals are rather open to emotions, which they regulate only when these are deemed problematic in a particular context. In view of this consideration, we are forced to conclude that high EI individuals' emotion regulatory processes cannot be fully automatic. Further research is thus needed to understand which stimuli/contexts give rise to automatic vs. effortful emotion regulation, as well as the moment when automatic processes take place (too early in the case of overly controlled individuals, too late or never in low EI individuals?).

### Long-term mental and physical health consequences

We have provided some evidence that high EI individuals are open to emotions and leave room for their emergence. Thus, they do not *always* regulate their emotions, but appear to know how to do so efficiently when necessary. Although findings reported so far seem to associate EI with the wise utilization of emotion regulation, the ultimate evidence in favor of the adaptive nature of EI would be to show that it correlates with superior indicators of adaptation, such as better mental and physical health.

A recent meta-analysis of the relationships between EI and health (105 effect sizes, 19,815 participants; by Martins et al., 2010) showed that EI was positively associated with mental health (*r* = 0.36) and self-reported physical health (*r* = 0.27). Mikolajczak et al. (in press) recently confirmed the positive association between trait EI and health using objective indicators of physical health available from participants' Mutual Benefit Society records (i.e., doctor consultations, hospitalization, drug consumption) (*r* = 0.11). This relationship between trait EI and physical health is not surprising as mounting evidence suggests that different emotion regulation strategies exert a distinct influence on physical health (Gross, 2013). For instance, researchers found that reappraisal was associated with lower levels of C-reactivity protein, whereas suppression was associated with higher levels of C-reactivity protein (Appleton et al., 2013). In another study, Kubzansky et al. (2011) found that self-regulation (understood as a psychological asset that enables individuals to manage feelings, thoughts, impulses, and behavior, with the capacity to regulate emotions serving as a central component) predicted a decreased subsequent risk of heart attacks and coronary heart disease in men, even when controlling for traditional coronary risk factors.

Several other factors may explain this relationship between EI and *physical* health. First, if high EI individuals regulate their emotions at the earliest possible point, they should experience less prolonged arousal in response to a negative situation/stimuli and thus be protected vis-à-vis the deleterious consequences of chronic arousal on physical health (e.g., coronary heart disease, gastro-intestinal disorders, asthma, psoriasis, migraine etc; Chrousos, 2000; Thurin and Baumann, 2003). Second, if high EI people do not need alcohol and drugs to anesthetize unwanted feelings, they should be consequently less at risk for substance-use related health problems such as cirrhosis of the liver, pancreatitis, and polyneuropathy. Third, their increased emotion regulation efficiency should have a positive effect on their sleep, which is known to be crucial to health (Belloc and Breslow, 1972; Pilcher and Ott, 1998; Gottlieb et al., 2006). Brown and Schutte (2006) indeed showed that higher trait EI is associated with better quality and more refreshing sleep.

### Regulation strategies vs. regulation styles

Authors in the ER tradition have repeatedly warned against classifying strategies as irremediably (i.e., always) good or bad (Barrett and Gross, 2001; Gross and Thompson, 2007). They argue that the efficiency of a strategy depends on the context and the angle from which we see things (Aldao et al., 2015).

To illustrate, we can take the example of a man who prides himself on being strong and independent, and thus suppresses his anger and sadness when his girlfriend confesses that she cheated on him. The success or value of this strategy depends on the criteria adopted. In this case, his reaction could be deemed successful with respect to the regulator's goals (the strategy used allows him to meet his goal: appearing strong and independent in the eyes of his girlfriend), with respect to some social norms (i.e., his reaction is aligned with social norms concerning avoidance of unmanly reactions such as crying and violent reactions such as hitting), and with respect to the relationship from a short-term perspective (the strategy used makes it possible to avoid a fight). This same strategy could be deemed unsuccessful with respect to the relationship from a long-term perspective (his girlfriend does not get a chance to know how hurt he really is. She might therefore cheat on him again, leading eventually to a breakup), and possibly with respect to the man's long-term adaptation (by hiding his emotions, he prevents people from sensing his needs and exposes himself to further negative emotions, thereby endangering his long-term well-being and health).

Whereas we believe that it is crucial to understand that a given emotion regulation *strategy* cannot be considered as adaptive or maladaptive *per se*—namely, irrespective of the context, the temporal perspective, and the individual's goals—this review nonetheless supports the idea that different emotion regulation *styles* (i.e., the repeated use of a given emotion regulation pattern) carry different consequences for longer-term adaptation. Inasmuch as EI predicts successful adaptation in a number of domains (e.g., work performance, marital/social relationships), the fact that it is positively associated with some strategies (e.g., problem-focused coping, reappraisal) and negatively with others (e.g., rumination, substance use) suggests that some regulation *styles* are more adaptive than others. This view is consistent with preliminary empirical evidence obtained in the emotion regulation tradition (e.g., Gross and John, 2003; John and Gross, 2007) and the accumulated evidence in coping research (e.g., Zeidner and Endler, 1996) and in other specific domains (e.g., Nolen-Hoeksema et al., 1993 regarding rumination).

### Real-world consequences of emotion regulation families

The foregoing theoretical consideration suggests that research in the ER tradition would benefit from delving more deeply into the investigation of the consequences of different emotion regulation styles for adaptation. For instance, what are the relative costs and benefits of each regulation family? Aldao et al.'s 2010) meta-analysis showed that some ER strategies are most represented in psychopathological groups, thereby suggesting that these may be less effective. The meta-analysis carried out by Webb et al. (2012) nicely complemented these findings by comparing the relative efficiency of the various ER strategies regarding experimentally-induced emotions. A meta-analysis that could compare the relative short- and long-term efficiency of ER strategies regarding real-life events is needed to complete the picture, but the current paper provides a preliminary idea of what the findings may look like (at least in the long-term perspective).

We further believe that the real-world contexts explored by EI researchers constitute a crucial testing-ground for the predictions made in the ER tradition. Gross (2002) and Gross and John (2003) have investigated the social consequences of the use of reappraisal and suppression but this work should be extended to other regulation strategies and other domains of life. One important issue to be addressed concerns the outcomes of different emotion regulation styles in family, friendship, educational, and work settings. For instance, do people who chronically use reappraisal have better social and marital relationships? Are they more efficient at work than those who use distraction or emotion expression? Another issue that needs to be addressed concerns the *nature* of the benefits/costs of each regulation style. Indeed, as we have emphasized above, strategies can be differentially successful depending on the criteria adopted (short term vs. long term, self vs. other vs. the relationship, emotion regulation vs. task efficiency). Accordingly, it is conceivable that reappraisal and distraction are equally efficient in decreasing the negative affect induced by a given task, but that the former is more efficient than the latter regarding one's performance of the task. Studies such as these are needed to determine whether some strategies are more successful than others in maximizing adaptation in a large number of domains.

Finally, it is possible that the key to successful adaptation lies not only in the use of effective strategies but also in the breadth of one's ER repertoire and in the flexibility with which one uses these regulation strategies. Studies are urgently needed to address strategic flexibility, namely the ability to modify one's regulation style when it is not appropriate for the situation at hand (Bonanno et al., 2004; Aldao et al., 2015). Strategic flexibility seems indeed essential for one's adaptation to a complex and changing environment such as ours, in which a usually functional regulation style can at times turn out to be dysfunctional (e.g., the rigid utilization of a usually functional regulation style such as problem-focused coping can have serious costs in uncontrollable situations such as an incurable disease).

## Concluding comment

In this article, we have drawn together two relatively independent research traditions that both capture an important aspect of emotion management. The ER tradition has shed light on emotion regulation processes while the EI tradition has documented the consequences of individual differences in emotion regulation on social, health, educational and work outcomes.

The goal of the present paper was to use the ER conceptual framework (i.e., the process model of emotion regulation) to characterize and organize the emotion regulation processes underlying the construct of emotional intelligence. The benefit for the EI tradition is an enhanced understanding of *why* high levels of EI are associated with better outcomes, whereas the benefit for the ER tradition is a better understanding of the social, health, educational, and occupational consequences of certain emotion regulation styles.

Although much research remains to be done to clarify the relationship between emotional intelligence and emotion regulation, the present paper suggests that EI is a useful construct to capture individual differences in emotion regulation. This probably explains why trait EI has demonstrated incremental validity to predict emotion-related processes and outcomes over and above the five-factor model of personality in many studies (e.g., Petrides and Furnham, 2003; Petrides et al., 2006a, 2007a; Mikolajczak et al., 2007a,c). It is our hope that this article will help to bridge the gap between ER and EI traditions and stimulate research on individual differences in emotion regulation processes and their real-world consequences.

## Author note

Preparation of this paper was facilitated by a pre-doctoral research grant from the Basque Government, a post-doctoral research grant from the Belgian National Fund for Scientific Research (FNRS) and a post-doctoral travel grant from the Université catholique de Louvain to the second author, and NIH Grant R01 MH58147 to the third author.

### Conflict of interest statement

The authors declare that the research was conducted in the absence of any commercial or financial relationships that could be construed as a potential conflict of interest.

## Footnotes

^1^Mayer Salovey Caruso Emotional Intelligence Test (Mayer et al., 2002).

^2^Situational Test of Emotional Understanding (MacCann and Roberts, 2008).

^3^Geneva Emotion Recognition Test (Schlegel et al., 2014).

^4^Trait Emotional Intelligence Questionnaire (Petrides, 2009).

^5^Emotional Quotient Inventory (Bar-On, 2004).

^6^Profile of Emotional Competence (Brasseur et al., 2013).

^7^The authors provided us with subcorrelations between Trait EI and the “Behavioral Disengagement” subscale of the COPE.

^8^See footnote 7.

^9^The authors provided us with subcorrelations between Trait EI and the “Active Coping” and “Planning Coping” subscales of the COPE.

^10^The authors provided us with subcorrelations between Trait EI and the “Restraint Coping” subscale of the COPE.

^11^The authors provided us with subcorrelations between Trait EI and the “Instrumental Social Support” subscale of the COPE.

^12^The authors provided us with subcorrelations between Trait EI and the “Active Coping” and “Planning Coping” subscales of the COPE.

^13^The authors provided us with subcorrelations between Trait EI and the “Restraint Coping” subscale of the COPE.

^14^The authors provided us with subcorrelations between Trait EI and the “Instrumental Social Support” subscale of the COPE.

^15^The authors provided us with subcorrelations between Trait EI and the “Mental Disengagement” subscale of the COPE.

^16^See footnote 15.

^17^The terms initial appraisal and reappraisal are used here for conceptual clarity. In practice, however, it is difficult to distinguish initial appraisal from reappraisal because appraisals are not a single perception emerging directly after encountering the potentially stressful situation then stopping. Rather, appraisals are continuously updated as the situation and one's own actions/feelings change over time.

^18^The authors provided us with subcorrelations between Trait EI and the “Positive Reinterpretation” subscale of the COPE.

^19^The authors provided us with subcorrelations between Trait EI and the “Denial” subscale of the COPE.

^20^The authors provided us with subcorrelations between Trait EI and the “positive reappraisal” and “Humour” subscales of the COPE.

^21^The authors provided us with subcorrelations between Trait EI and the “Acceptance” subscale of the COPE.

^22^See footnote 19.

^23^The authors provided us with subcorrelations between Trait EI and the “positive reappraisal,” “acceptance,” and “Humour” subscales of the COPE.

^24^The authors provided us with subcorrelations between Trait EI and the “Venting” subscale of the COPE.

^25^Emotional labor refers to the act of managing emotions and emotional expressions in order to be consistent with organizational “display rules,” defined as the organizationally required emotions during interpersonal service transactions (Hochschild, 1983).

^26^The authors provided us with subcorrelations between Trait EI and the “Alcohol/Drug Use” subscale of the COPE.

^27^The authors provided us with subcorrelations between Trait EI and the “Venting” subscale of the COPE.

^28^See footnote 26.
