# Corrigendum: Integrating emotion regulation and emotional intelligence traditions: a meta-analysis

**DOI:** 10.3389/fpsyg.2019.02610

**Published:** 2019-11-27

**Authors:** Ainize Peña-Sarrionandia, Moïra Mikolajczak, James J. Gross

**Affiliations:** ^1^Faculty of Psychology, Department of Personality, Evaluation and Psychological Treatments, University of the Basque Country, Donostia-San Sebastian, Spain; ^2^Department of Psychology, Research Unit for Emotion Cognition and Health, Université Catholique de Louvain, Louvain-la-Neuve, Belgium; ^3^Department of Psychology, Stanford University, Stanford, CA, United States

**Keywords:** emotional intelligence, emotional competence, emotion regulation, coping, review, meta-analysis

In the original article, there was a mistake in Table 1 as published. In Goldenberg's study, measuring the relationship between ability EI and social support, *r* appears as 0.42, CI (lower bound) as 0.31, CI (upper bound) as 0.52, and *d* as 0.92. However, *r* should be 0.04, CI (lower bound) −0.09, CI (upper bound) 0.17, and *d* 0.08. The corrected Table 1 appears below.

**Table 1:** Linking emotional intelligence to the use of emotion regulation strategies.

**Authors**	***N***	**ER Family**	**Specific variable**	**T/A**	**Measure used**	**Facet**	***r***	**CI *r* (lower bound)**	**CI *r* (upper bound)**	***d***
Schutte et al., 2009	73	SS	Situation selection	T	AES		0.30	0.08	0.49	0.63
Ciarrochi et al., 2002	302	SS	Daily hassles	T	EIS		−0.01	−0.12	0.10	−0.02
Day et al., 2005	133	SS	Daily hassles	T	EQ-I	AGS	−0.43	−0.51	−0.27	−0.94
Kim and Agrusa, 2011	385	SS	Avoidant coping	T	WLEIS		0.15	0.05	0.25	0.30
Shah and Thingujam, 2008	197	SS	Avoidant coping	T	EIS	AGS	−0.08	−0.22	0.06	−0.16
Rogers et al., 2006	253	SS	Avoidant coping	T	SREIT		−0.04	−0.16	0.08	−0.08
Velasco et al., 2006	593	SS	Avoidant coping	T	TMMS-alex F1		−0.16	−0.24	−0.08	−0.32
Petrides et al., 2007a (study 1)	166	SS	Avoidant coping	T	modified EQ-i		−0.34	−0.47	−0.20	−0.72
Petrides et al., 2007a (study2 sample1)	200	SS	Avoidant coping	T	TEIQue-LF		−0.39	−0.50	−0.27	−0.84
Petrides et al., 2007b	274	SS	Avoidant coping	T	TEIQue-LF		0.01	−0.11	0.13	0.02
MacCann et al., 2011	159	SS	Avoidant coping	A	MSCEIT	AGS	−0.21	−0.35	−0.06	−0.43
Gerits et al., 2004	380	SS	Avoidant coping	T	EQ-i		−0.23	−0.32	−0.13	−0.47
Monaci et al., 2013	198	SS	Avoidant coping	T	SEIS		0.01	−0.13	0.15	0.02
Mikolajczak et al., 2009a	490	SS	Avoidant coping	T	TEIQue-ASF		−0.26	−0.34	−0.18	−0.54
Petrides et al., 2006a	37	SS	Perseverance	T	TEIQue-LF		0.53	0.25	0.74	1.25
Dunn et al., 2007	84	SS	Forecast accuracy	A	MSCEIT		−0.19	0.06	0.62	−0.38
Dunn et al., 2007	84	SS	Forecast accuracy	T	SREIS		−0.09	−0.39	0.02	−0.18
Hoerger et al., 2012 (study 1)	81	SS	Forecast accuracy	T	SEI/SREIS/TEIQue		0.22	−0.30	0.13	0.45
Hoerger et al., 2012 (study 1)	81	SS	Forecast accuracy	A	JET+IJI		0.36	−0.01	0.41	0.77
Hoerger et al., 2012 (study 2)	81	SS	Forecast accuracy	T	TEIQue-SF		0.27	0.16	0.54	0.56
Hoerger et al., 2012 (study 2)	81	SS	Forecast accuracy	A	JET+IJI+STEU		0.39	0.07	0.47	0.84
Schutte et al., 2001b	38	SS	Persistence	T	SEIS		0.37	0.19	0.56	0.79
Bastian et al., 2005	246	SS	Behavioral disengagement	T	TMMS+AES	R+C	−0.24	−0.35	−0.12	−0.49
Bastian et al., 2005	246	SS	Behavioral disengagement	A	MSCEIT		−0.16	−0.28	−0.04	−0.32
Tsaousis and Nikolaou, 2005	365	SS	Time to relax	T	TEIQ		0.43	0.35	0.51	0.95
Schutte et al., 2009	73	SM	Modifying situations	T	EIS		0.20	−0.03	0.42	0.41
Moradi et al., 2011	200	SM	Problem-focused coping	T	TMMS	R+C	0.27	0.14	0.40	0.56
Petrides et al., 2007a (study 1)	166	SM	Problem-focused coping	T	modified EQ-i		0.57	0.46	0.67	1.38
Petrides et al., 2007a (study 2. sample 1)	200	SM	Problem-focused coping	T	TEIQue-LF		0.67	0.59	0.75	1.80
Petrides et al., 2007b	274	SM	Problem-focused coping	T	TEIQue-LF		0.64	0.57	0.71	1.66
MacCann et al., 2011	159	SM	Problem-focused coping	A	MSCEIT	AGS	0.14	−0.02	0.29	0.28
Shah and Thingujam, 2008	197	SM	Problem-focused coping	T	EIS	AGS	0.21	0.07	0.34	0.43
Goldenberg et al., 2006	223	SM	Problem-focused coping	T	SREIS		0.55	0.46	0.64	1.31
Goldenberg et al., 2006	223	SM	Problem-focused coping	A	MSCEIT		0.17	0.04	0.30	0.34
Kluemper, 2008	180	SM	Problem-focused coping	T	WLEIS		0.61	0.51	0.70	1.54
Rogers et al., 2006	253	SM	Problem-focused coping	T	SREIT		0.30	0.19	0.41	0.63
Mikolajczak et al., 2008	203	SM	Problem-focused coping	T	TEIQue-LF		0.40	0.29	0.51	0.87
Salovey et al., 2002 (study 3)	48	SM	Problem-focused coping	T	TMMS	R+C	0.34	0.06	0.57	0.71
Saklofske et al., 2007	362	SM	Problem-focused coping	T	EIS		0.38	0.29	0.46	0.82
Almran and Punamaki, 2008	312	SM	Problem-focused coping	T	EQ-I:YV-S		0.25	0.14	0.36	0.51
Velasco et al., 2006	593	SM	Problem-focused coping	T	TMMS-alex F1		0.20	0.12	0.28	0.41
Rahim and Minors, 2003	222	SM	Problem solving	T	EQ-Index	AGS	0.44	0.33	0.54	0.98
Monaci et al., 2013	198	SM	Direct confrontation	T	SEIS		0.43	0.31	0.54	0.95
Montes-Berges and Augusto, 2007	119	SM	Active coping	T	TMMS-24	R+C	0.05	−0.13	0.23	0.11
Gerits et al., 2004	380	SM	Active coping	T	EQ-I		0.38	0.29	0.46	0.82
Tsarenko and Strizhakova, 2013	252	SM	Active coping	T	SREIS		0.45	0.34	0.54	1.01
Zomer, 2012	300	SM	Active coping	T	TMMS-24	R+C	0.15	0.04	0.26	0.30
Bastian et al., 2005	246	SM	Active coping + planning	T	TMMS+AES	R+C	0.40	0.28	0.51	0.87
Bastian et al., 2005	246	SM	Active coping + planning	A	MSCEIT		0.05	−0.08	0.18	0.10
Bastian et al., 2005	246	SM	Problem Solving Inventory	T	TMMS+AES	R+C	−0.43	−0.53	−0.32	−0.94
Bastian et al., 2005	246	SM	Problem Solving Inventory	A	MSCEIT		−0.04	−0.17	0.09	−0.08
Austin et al., 2010	475	SM	Task-oriented coping	T	EQ:i-S	AGS	0.38	0.30	0.45	0.82
Saklofske et al., 2012	238	SM	Task-oriented coping	T	EQ:i S	AGS	0.48	0.38	0.57	1.09
Kim and Agrusa, 2011	385	SM	Task coping	T	WLEIS		0.54	0.46	0.61	1.28
Mikolajczak et al., 2009a	490	SM	Rational coping	T	TEIQue-ASF		0.46	0.39	0.53	1.03
Monaci et al., 2013	198	SM	Social support	T	SEIS		0.36	0.24	0.48	0.77
Bastian et al., 2005	246	SM	Instrumental social support	T	TMMS+AES	R+C	0.24	0.11	0.35	0.49
Bastian et al., 2005	246	SM	Instrumental social support	A	MSCEIT		0.06	−0.07	0.19	0.12
Zomer, 2012	300	SM	Support from others	T	TMMS-24	R+C	0.11	0.00	0.22	0.21
Gerits et al., 2004	380	SM	Social support seeking	T	EQ-i		0.21	0.11	0.31	0.43
Shah and Thingujam, 2008	197	SM	Social support seeking	T	EIS	AGS	0.07	−0.08	0.21	0.13
Ciarrochi and Deane, 2001	300	SM	Social support seeking	T	EIS	AGS	0.15	0.05	0.25	0.26
Velasco et al., 2006	593	SM	Social support seeking	T	TMMS-alex F1		0.13	0.05	0.21	0.26
Goldenberg et al., 2006	223	SM	Social support seeking	T	SREIS		0.32	0.20	0.43	0.67
Goldenberg et al., 2006	223	SM	Social support seeking	A	MSCEIT		0.04	−0.09	0.17	0.08
Zeidner and Kloda, 2013	200	SM	Conflict res. (constructive)	A	MSCEIT		0.24	0.11	0.37	0.49
Zeidner and Kloda, 2013	200	SM	Conflict res. (avoidance)	A	MSCEIT		−0.39	−0.50	−0.26	−0.84
Jordan and Troth, 2004	350	SM	Conflict res. (integrate)	T	WEIP6		0.35	0.25	0.44	0.74
Jordan and Troth, 2004	350	SM	Conflict res. (avoid)	T	WEIP6		−0.12	−0.23	−0.01	−0.24
Jordan and Troth, 2004	350	SM	Conflict res. (dominate)	T	WEIP6		0.19	0.09	0.29	0.38
Jordan and Troth, 2002	139	SM	Conflict res. (collaborate)	T	WEIP6		0.53	0.40	0.64	1.25
Jordan and Troth, 2002	139	SM	Conflict res. (avoidance)	T	WEIP6		−0.12	−0.28	0.04	−0.24
Jordan and Troth, 2002	139	SM	Conflict resolution (force)	T	WEIP6		0.02	−0.15	0.19	0.04
Jordan and Troth, 2002	139	SM	Conflict res. (accommodate)	T	WEIP6		−0.08	−0.24	0.08	−0.01
Jordan and Troth, 2002	139	SM	Conflict res. (compromise)	T	WEIP6		0.09	−0.08	0.25	0.02
Salami, 2010b	320	SM	Conflict res. (confronting)	T	WLEIS		0.20	0.10	0.30	0.41
Salami, 2010b	320	SM	Conflict res. (withdrawal)	T	WLEIS		0.12	0.01	0.23	0.24
Salami, 2010b	320	SM	Conflict resolution (forcing)	T	WLEIS		0.12	0.01	0.23	0.24
Salami, 2010b	320	SM	Conflict res. (smoothe)	T	WLEIS		0.19	0.08	0.29	0.38
Salami, 2010b	320	SM	Conflict res. (compromise)	T	WLEIS		0.21	0.10	0.31	0.43
Bastian et al., 2005	246	SM	Restraint	T	TMMS+AES		0.08	−0.05	0.21	0.16
Bastian et al., 2005	246	SM	Restraint	A	MSCEIT		−0.04	−0.17	0.09	−0.08
Schutte et al., 2009	73	AD	Attention deployment	T	EIS		0.38	0.17	0.56	0.82
Bastian et al., 2005	246	AD	Mental disengagement	T	TMMS+AES		−0.05	−0.18	0.08	−0.10
Bastian et al., 2005	246	AD	Mental disengagement	A	MSCEIT		−0.05	−0.18	0.08	−0.10
Mikolajczak et al., 2008	203	AD	Trait distraction	T	TEIQue-LF		0.41	0.29	0.52	0.89
Salovey et al., 2002 (study 3)	48	AD	State distraction	T	TMMS	R+C	0.36	0.08	0.59	0.77
Saklofske et al., 2012	238	AD	Distraction	T	EQ-i: S		−0.11	−0.23	0.02	−0.21
Austin et al., 2010	475	AD	Distraction	T	EQ-i: S		0.18	0.09	0.27	0.37
Lanciano et al., 2012	157	AD	Dysfunctional rumination	A	MSCEIT	AGS	−0.44	−0.56	−0.30	−0.98
Petrides et al., 2007b	274	AD	Trait rumination	T	TEIQue-LF		−0.47	−0.56	−0.38	−1.06
Petrides et al., 2007a (study 1)	166	AD	Trait rumination	T	modified EQ-i		−0.53	−0.64	−0.42	−1.25
Mikolajczak et al., 2008	203	AD	Trait Rumination	T	TEIQue-LF		−0.10	−0.24	0.04	−0.20
Ramos et al., 2007	144	AD	State rumination	T	TMMS	R+C	−0.11	−0.27	0.05	−0.21
Salovey et al., 2002 (study 3)	48	AD	State rumination	T	TMMS	R+C	−0.27	−0.51	0.02	−0.55
Salguero et al., 2013	1154	AD	Rumination	T	TMMS-24	R+C	−0.11	−0.17	−0.05	−0.22
Brown and Ryan, 2003	645	AD	Mindful attention	T	TMMS-24	R+C	0.40	0.33	0.46	0.87
Wang and Kong, 2014	321	AD	Mindful attention	T	WLEIS		0.33	0.23	0.42	0.70
Schutte and Malouff, 2011	125	AD	Mindful attention	T	AES		0.65	0.53	0.74	1.71
Kokinda, 2010	108	AD	Mindful attention	T	AES		0.47	0.31	0.63	1.06
Charoensukmongkol, 2014	317	AD	Mindful attention	T	WLEIS		0.32	0.22	0.42	0.68
Baer et al., 2004 (study 4)	130	AD	Mindful attention	T	TMMS-24	R+C	0.24	0.07	0.40	0.49
Totterdell and Holman, 2003	18	AD	Focus on positive	T	EIS		0.46	−0.01	0.79	1.03
Schutte et al., 2009	73	CC	Cognitive change	T	AES		0.26	0.03	0.46	0.54
Charoensukmongkol, 2014	317	CC	Self-efficacy	T	WLEIS		0.68	0.61	0.74	1.86
Brown et al., 2003	288	CC	Self-efficacy	T	EII-R		0.25	0.14	0.36	0.50
Chan, 2004	158	CC	Self-efficacy	T	EIS		0.32	0.17	0.45	0.66
Martin et al., 2004	140	CC	Self-efficacy	T	EJI		0.54	0.41	0.65	1.27
Durán et al., 2006	373	CC	Self-efficacy	T	TMMS	R+C	0.34	0.24	0.42	0.72
Kaur et al., 2006	117	CC	Self-efficacy	T	EIS		0.42	0.26	0.56	0.92
Villanueva and Sanchez, 2007	70	CC	Self-efficacy	T	SSRI		0.36	0.13	0.54	0.77
Di Fabio and Palazzeschi, 2008	169	CC	Self-efficacy	T	EQ-i		0.34	0.20	0.47	0.72
Adeyemo, 2007	300	CC	Self-efficacy	T	EQ-i		0.17	0.06	0.28	0.34
Mikolajczak et al., 2006	95	CC	Self-efficacy	T	TEIQue-LF		0.66	0.53	0.76	1.75
Mikolajczak and Luminet, 2008 (study1)	27	CC	Self-efficacy	T	TEIQue-LF		0.50	0.15	0.75	1.15
Mikolajczak and Luminet, 2008 (study2)	15	CC	Self-efficacy	T	TEIQue-SF		0.29	−0.27	0.70	0.60
Kirk et al., 2008	207	CC	Self-efficaccy	T	AES		0.73	0.66	0.79	2.13
Kirk et al., 2008	207	CC	Self-efficaccy	A	MSCEIT		0.34	0.22	0.46	0.72
Animasahun, 2008	300	CC	Self-efficacy	T	EIS		0.43	0.33	0.52	0.95
Salami, 2010a	242	CC	Self-efficacy	T	WLEIS		0.08	−0.05	0.21	0.16
Tsarenko and Strizhakova, 2013	252	CC	Self-efficacy	T	SREIS		0.57	0.48	0.65	1.38
Mouton et al., 2013	119	CC	Self-efficacy	T	TEIQue		0.28	0.11	0.44	0.58
Di Fabio and Saklofske, 2014b	164	CC	Core self-evaluation^a^	T	TEIQue/EQ-i		0.56	0.45	0.65	1.35
Di Fabio and Saklofske, 2014b	164	CC	Core self-evaluation	A	MSCEIT		0.02	−0.13	0.17	0.04
Kluemper, 2008	180	CC	Core self-evaluation	T	WLEIS		0.73	0.65	0.79	2.14
Di Fabio and Saklofske, 2014a	194	CC	Self-Efficacy	A	MSCEIT		0.23	0.09	0.35	0.47
Di Fabio and Saklofske, 2014a	194	CC	Self-Efficacy	T	EQ-i		0.67	0.58	0.74	1.80
Mikolajczak and Luminet, 2008 (study2)	15	CC	Ratio challenge/threat	T	TEIQue-SF		0.72	0.33	0.9	2.07
Mikolajczak et al., 2006	70	CC	Challenge appraisal	T	TEIQue-LF		0.02	−0.22	0.26	0.04
Mikolajczak et al., 2006	70	CC	Threat appraisal	T	TEIQue-LF		−0.41	−0.60	−0.20	−0.89
Schutte et al., 2009	73	CC	Reappraisal	T	AES		0.46	0.26	0.62	1.03
Shah and Thingujam, 2008	197	CC	Positive reappraisal	T	EIS	AGS	0.21	0.07	0.34	0.43
Mikolajczak et al., 2008	203	CC	Reappraisal	T	TEIQue-LF		0.46	0.34	0.56	1.03
Velasco et al., 2006	593	CC	Positive reappraisal	T	TMMS-alex F1		0.23	0.15	0.31	0.47
Kafetsios and Loumakou, 2007	475	CC	Reappraisal	T	EQ-I		0.04	−0.08	0.16	0.08
Cabello et al., 2013	866	CC	Reappraisal	T	TMMS-24	R+C	0.34	0.28	0.40	0.72
Coumans, 2005	31	CC	Reappraisal	T	TEIQue-LF		0.02	−0.34	0.38	0.04
Totterdell and Holman, 2003	18	CC	Perspective taking	T	EIS		0.21	−0.29	0.62	0.43
Moradi et al., 2011	200	CC	Reappraisal	T	TMMS	R+C	0.45	0.34	0.56	1.01
Bastian et al., 2005	246	CC	Positive interpretation	T	TMMS+AES	R+C	0.50	0.40	0.59	1.15
Bastian et al., 2005	246	CC	Positive interpretation	A	MSCEIT		0.11	−0.02	0.24	0.21
Bastian et al., 2005	246	CC	Adaptive Humour	T	TMMS+AES	R+C	0.14	0.01	0.26	0.28
Bastian et al., 2005	246	CC	Adaptive Humour	A	MSCEIT		0.04	−0.09	0.17	0.08
Zomer, 2012	300	CC	Adaptive Humour	T	TMMS-24	R+C	0.01	−0.10	0.12	0.02
Greven et al., 2008	1038	CC	Adaptive Humour	T	TEIQue-LF		0.45	0.40	0.50	0.99
Greven et al., 2008	1038	CC	Maladaptive Humour	T	TEIQue-LF		−0.27	−0.33	−0.21	−0.55
Tsarenko and Strizhakova, 2013	252	CC	Denial	T	SREIS		−0.09	−0.21	0.03	−0.18
Zomer, 2012	300	CC	Denial	T	TMMS-24	R+C	−0.16	−0.27	−0.05	−0.32
Bastian et al., 2005	246	CC	Denial	T	TMMS+AES		−0.15	−0.27	−0.02	−0.30
Bastian et al., 2005	246	CC	Denial	A	MSCEIT		−0.20	−0.32	−0.08	−0.41
Zomer, 2012	300	CC	Acceptance	T	TMMS-24	R+C	0.09	−0.02	0.20	0.17
Bastian et al., 2005	246	CC	Acceptance	T	TMMS+AES		0.38	0.26	0.48	0.82
Bastian et al., 2005	246	CC	Acceptance	A	MSCEIT		0.10	−0.03	0.23	0.21
Mikolajczak et al., 2008	203	CC	Acceptance	T	TEIQue-LF		−0.06	−0.20	0.08	−0.12
Bastian et al., 2005	246	RM	Venting	T	TMMS+AES		0.05	−0.08	0.18	0.10
Bastian et al., 2005	246	RM	Venting	A	MSCEIT		0.02	−0.11	0.15	0.03
Zomer, 2012	300	RM	Venting	T	TMMS-24	R+C	−0.27	−0.37	−0.16	−0.56
Schutte et al., 2009	73	RM	suppression (ERQ)	T	EIS		−0.50	−0.66	−0.31	−1.15
Johnson and Spector, 2007	176	RM	Suppression	T	WLEIS		−0.08	−0.23	0.07	−0.16
Austin et al., 2008	247	RM	Suppression	T	TEIQue-SF		−0.45	−0.55	−0.35	−1.01
Mikolajczak et al., 2007b	124	RM	Suppression	T	TEIQue-SF		−0.31	−0.46	−0.14	−0.65
Totterdell and Holman, 2003	18	RM	Suppression	T	EIS		−0.18	−0.60	0.31	−0.36
Velasco et al., 2006	593	RM	Suppression	T	TMMS-alex F1		−0.28	−0.35	−0.21	−0.58
Kafetsios and Loumakou, 2007	475	RM	Suppression (ERQ)	T	EQ-I		−0.08	−0.20	0.04	−0.16
Cabello et al., 2013	866	RM	Suppression	T	TMMS-24	R+C	−0.13	−0.20	−0.06	−0.26
Lee and Ok, 2012	309	RM	Emotional dissonance	T	WLEIS		−0.22	−0.33	−0.11	−0.45
Rivers et al., 2013	243	RM	Aggressive behavior	A	MSCEIT		−0.25	−0.36	−0.13	−0.51
Brackett et al., 2004	330	RM	Deviant behavior	A	MSCEIT		−0.27	−0.37	−0.17	−0.56
Brackett and Mayer, 2003	207	RM	Social deviance	T	EQ-i/SREIT		−0.14	−0.27	0.00	−0.28
Shahzad et al., 2013	140	RM	Aggression	T	TEIQue-ASF		−0.31	−0.45	−0.15	−0.64
Mikolajczak et al., 2009a	490	RM	Self-harm	T	TEIQue-ASF		−0.31	−0.39	−0.23	−0.65
Karim and Shah, 2014	192	RM	Suicidal ideation	A	MSCEIT	AGS	−0.30	−0.42	−0.16	−0.63
Aradilla-Herrero et al., 2014	93	RM	Suicide risk	T	TMMS-24	R+C	−0.21	−0.40	−0.01	−0.43
Gardner et al., 2014	235	RM	Bulimic symptoms	T	MEIA		−0.22	−0.34	−0.10	−0.45
Gardner et al., 2014	235	RM	Bulimic symptoms	A	MSCEIT		−0.07	−0.20	0.06	−0.14
Gardner et al., 2014	235	RM	Binge eating	T	MEIA		−0.21	−0.33	−0.08	−0.43
Gardner et al., 2014	235	RM	Binge eating	A	MSCEIT		−0.03	−0.16	0.10	−0.06
Pettit et al., 2010	402	RM	Bulimia/Food preocupation	T	TMMS-24	R+C	−0.13	−0.23	−0.03	−0.26
Markey and Vander Wal, 2007	154	RM	Bulimic symptoms	T	EQ- i:S		−0.31	−0.45	−0.16	−0.65
Brackett et al., 2004	330	RM	Illegal drug use	A	MSCEIT		−0.11	−0.22	0.00	−0.22
Brackett et al., 2004	330	RM	Alcohol use	A	MSCEIT		−0.13	−0.24	−0.02	−0.26
Rossen and Kranzler, 2009	150	RM	Alcohol use	A	MSCEIT		−0.21	−0.35	−0.07	−0.43
Tsaousis and Nikolaou, 2005	365	RM	Alcohol units	T	TEIQ		−0.07	−0.17	0.03	−0.14
Austin et al., 2005	115	RM	Acohol consumption	T	REIS		−0.19	−0.37	−0.01	−0.38
Ghee and Johnson, 2008	214	RM	Alcohol consumption	T	EIS		−0.02	−0.15	0.11	−0.04
Riley and Schutte, 2003	141	RM	Acohol consumption	T	EIS		−0.34	−0.48	−0.2	−0.72
Brackett and Mayer, 2003	207	RM	Alcohol consumption	T	EQ-i/SREIT		−0.13	−0.26	0.01	−0.26
Trinidad and Johnson, 2002	205	RM	Alcohol consumption	A	MEIS		−0.12	−0.26	0.01	−0.25
Saklofske et al., 2007	362	RM	Alcohol consumption	T	EIS		−0.05	−0.15	0.05	−0.10
Brackett and Mayer, 2003	207	RM	Illegal drug user scale	T	EQ-i/SREIT		−0.14	−0.27	−0.003	−0.28
Riley and Schutte, 2003	141	RM	Drug abuse	T	EIS		−0.42	−0.55	−0.29	−0.92
Limonero et al., 2006	133	RM	Canabis smoking	T	TMMS	R+C	−0.01	−0.19	0.16	−0.02
Bastian et al., 2005	246	RM	Substance use	T	TMMS+AES		−0.05	−0.18	0.08	−0.10
Bastian et al., 2005	246	RM	Substance use	A	MSCEIT		−0.02	−0.14	0.11	−0.03
Rivers et al., 2013	243	RM	Substance abuse	A	MSCEIT		−0.18	−0.31	−0.06	−0.36
Schutte et al., 2011	100	RM	Alcohol problems	A	MSCEIT		−0.30	−0.46	−0.11	−0.63
Schutte et al., 2011	100	RM	Alcohol problems	T	AES		−0.27	−0.45	−0.08	−0.56
Zomer, 2012	300	RM	Drugs	T	TMMS-24	R+C	−0.11	−0.22	0.00	0.21
Monaci et al., 2013	198	RM	Alcohol use	T	SEIS		−0.05	−0.19	0.09	−0.10
Solanki and Lane, 2010	315	RM	Exercise mood regulating	T	EIS		0.45	0.35	0.53	1.01

*SS, Situation Selection; SM, Situation Modification; AD, Attentional Deployment; CC, Cognitive Change; RM, Response Modulation; T/A, Trait or Ability EI measure: T, trait EI measure, A, ability EI measure; AGS, Aggregated Global Score; R+C, Repair + Clarity; AES, Assessing Emotions Scale; EIS, Emotional Intelligence Scale; EQ-i/YV/S, Emotional Quotient Inventory/Young Version/Short; WLEIS, The Wong and Law Emotional Intelligence Scale; SREIT, Self Report Emotional Intelligence Test; TMMS-24, Trait Meta Mood Scale; TEIQ, The Traits Emotional Intelligence Questionnaire; TEIQue/SF/LF/ASF, Trait Emotional Intelligence Questionnaire/ Short-Form/Long-Form/Adolescent Short-Form; MSCEIT, The Mayer-Salovey-Caruso Emotional Intelligence Test; SEIS, Schutte Emotional Intelligence Scale; SREIS, Self-Reported Emotional Intelligence Scale; EQ-Index, Emotional Quotient Index; WEIP6, Workgroup Emotional Intelligence Profile-Version 6; EII-R, Emotional Intelligence Inventory Revised; MEIS, Multifactoral Emotional Intelligence Scale; EJI, Emotional Judgment Inventory; SSRI, Schutte Self-Report Inventory; MEIA, Multidimensional Emotional Intelligence Assessment; REIS, Revised Emotional Intelligence Scale; SEI, Survey of Emotional Intelligence. JET, Judgment of Emotions Test; IJI, Interpersonal Judgment Inventory; STEU, Situational Test of Emotional Understanding*.

^*^The forecast accuracy indices were calculated such that higher numbers indicate poorer accuracy.

a *The core self-evaluation construct is a fundamental part of self-evaluated values, efficacy and abilities. It includes self-esteem, self-efficacy, internal locus of control and absence of pessimism*.

As a consequence of the first mistake (*r* in Goldenberg's study was not 0.42, but 0.04), in the original article there was a mistake in Table 3 as published. The *d* related to Social support seeking was 0.50 in the original article. However, *d* should be 0.10 and CI around *d* should be 0.01 and 0.19. The corrected Table 3 appears below.

**Table 3:** Linking emotional intelligence (ability) to the use of emotion regulation strategies.

**ER family**	**ER strategy**	**Number of studies**	**Total *N***	**Dir. of effect**	**Effect-size (*d*)**	**95% Confidence Interval around *d***
Situation selection	Forecast accuracy^*^	1	84	–	−0.45	[−0.67; −0.23]
	Forecast accuracy	2	162	+	0.81	[0.65; 0.97]
	Avoidant coping	1	159	–	−0.43	[−0.59; −0.27]
	Behavioral disengagement	1	246	–	−0.32	[−0.44; −0.19]
Situation modification	Problem solving	3	628	+	0.23	[0.15; 0.31]
	Problem solving (negative)	1	246	–	−0.08	[−0.21; 0.05]
	Social support seeking	2	469	+	0.10	[0.01; 0.19]
	Conflict resolution	1	200	+	0.49	[0.35; 0.63]
	Conflict resolution (avoid)	1	200	–	−0.85	[−0.99; −0.71]
	Restraint	1	246	+	0.15	[0.02; 0.28]
Attentional deployment	Rumination	1	157	–	−0.98	[−1.14; −0.82]
	Mental disengagement	1	246	–	−0.10	[−0.23; 0.03]
Cognitive change	Self-efficacy	3	564	+	0.47	[0.33; 0.61]
	Positive interpretation	1	246	+	0.21	[0.08; 0.34]
	Humour	1	246	+	0.08	[−0.05; 0.21]
	Denial	1	246	–	−0.41	[−0.54; −0.28]
	Acceptance	1	246	+	0.21	[0.08; 0.34]
Response modulation	Venting	1	246	+	0.27	[0.14; 0.40]
	Aggressive behavior	2	573	–	−0.54	[−0.62; −0.46]
	Self-harm	1	192	–	−0.63	[−0.77; −0.49]
	Substance use	7	1604	–	−0.27	[−0.32; −0.22]
	Bulimia/Food preocupation	2	470	–	−0.10	[−0.19; −0.01]

* *The forecast accuracy indices were calculated such that higher numbers indicate poorer accuracy*.

Furthermore, in the original article, there was an error. Interpretations were made considering the wrong *r* (in Goldenberg's study).

A correction has been made to **Results** section, subsection **EI and Situation Modification**, paragraph five:

“Regarding ability EI, the results obtained are consistent with those that use trait measures. Higher ability EI is associated with greater use of problem-focused coping (Goldenberg et al., 2006; MacCann et al., 2011; but see Bastian et al., 2005,^12^ for non-significant results), although there is no significant relationship between an individual's ability to restrain him or herself (wait for the appropriate moment to act and avoid acting prematurely) and EI (Bastian et al., 2005)^13^. Ability EI also relates to more social support seeking, although effect sizes are nearly null (see Bastian et al., 2005,^14^; Goldenberg et al., 2006)”.

The authors apologize for this error and state that this does not change the scientific conclusions of the article in any way. The original article has been updated.

## Footnotes

^12^The authors provided us with subcorrelations between Trait EI and the “Active Coping” and “Planning Coping” subscales of the COPE.

^13^The authors provided us with subcorrelations between Trait EI and the “Restraint Coping” subscale of the COPE.

^14^The authors provided us with subcorrelations between Trait EI and the “Instrumental Social Support” subscale of the COPE.
